# Structural, Spectroscopic, and Docking Analysis of N,O‐Donor Ligand Metal Complex Nanoparticles With Hypolipidemic Effects via Lipoprotein Lipase Activation in High‐Fat Diet Mice

**DOI:** 10.1002/cbdv.202403003

**Published:** 2024-12-27

**Authors:** Sherif M. Abd El‐Hamid, Safa W. Aziz, Amira A. Mohamed, Mohammed S. El‐Gedamy, Ahmed E. Salem, Soha F. Mohammed, Wael A. Zordok, Adriano Sofo, Mohamed A. Sabry, Sadeek A. Sadeek, Hazem S. Elshafie

**Affiliations:** ^1^ Department of Medical Laboratories Technology College of Health and Medical Technologies Al‐Ayen Iraqi University (AUIQ) Thi‐Qar Iraq; ^2^ Department of Laboratory and Clinical Sciences College of Pharmacy University of Babylon Babylon Babil Iraq; ^3^ Department of Basic Science Zagazig Higher Institute of Engineering and Technology Zagazig Egypt; ^4^ Department of Clinical biochemistry and Molecular biology Urology and Nephrology Center Mansoura Egypt; ^5^ Department of Chemistry The Egyptian Mineral Resources Authority (EMRA) Cairo Egypt; ^6^ Department of Chemistry Faculty of Science Zagazig University Zagazig Egypt; ^7^ Department of Agricultural, Forestry, Food and Environmental Sciences University of Basilicata Potenza Italy; ^8^ Department of Medicinal Chemistry Faculty of Pharmacy Mansoura University Mansoura Egypt

**Keywords:** density functional theory (DFT), hypolipidemic, lipoprotein lipase, metal complexes, spectroscopy

## Abstract

New Cd(II), Zn(II), and Cu(II) chelates with cetirizine.2HCl (CETZ.2HCl) in incidence of 1,10 phenanthroline monohydrate (Phen.H_2_O) were synthesized in search of new biologically active compounds. The ligands and their chelates were described by 1H NMR, FT‐IR, elemental analysis, UV–vis spectrophotometry, thermal analyses, molar conductance, x‐ray diffraction (XRD), and magnetic‐susceptibility measurements. FT‐IR demonstrated that CETZ.2HCl is bonded with metal ions, as a monodentate via carboxylate oxygen atom and Phen.H_2_O chelated via two nitrogen atoms. The molar conductivity data showed that the complexes were nonelectrolytes, whereas XRD data supported that the compounds were crystalline. Density functional theory (DFT) was utilized to gain insight into the compounds' optimized design. The effects of CETZ.2HCl and the complexes on the activity of lipoprotein‐(L)‐lipase in mice were investigated. Unlike Cd(II) complex, all the other compounds exhibited significant increase in lipase activity, with reduction in triglycerides. Cu(II) and Zn(II) complexes showed robust hypolipidemic efficacy evidenced by lower levels of total cholesterol and low‐density lipoprotein (LDL), concomitant with higher levels of high‐density lipoprotein (HDL). Furthermore, Zn(II) complex was a safe alternative as it has a lower liver toxicity. Molecular docking demonstrated that Cu(II) and Zn(II) chelates exhibited greater affinities to lipase than the parent ligand. Finally, Cu(II) complex showed the highest antibacterial activity.

## Graphical abstract

Hypolipidemic influence of CETZ.2HCl‐(H1‐antihistamine) metal‐complexes on “C57Bl/6‐mice” fed high‐fat‐diet.

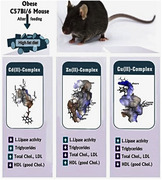

## 1 Introduction

A growing field of study in medical inorganic chemistry is being driven by the need for novel biologically active substances. Several studies have demonstrated that a medication's activity is increased when it is bound to a metallic element, and in certain circumstances, the complex has greater therapeutic benefits than the parent drug [1, 2, 3, 4, 5, 6, 7, 8]. At the turn of the 20th century, histamine was identified for the first time as a key moderator in allergic reactions [9]. H1 antihistamines were obtainable for over 50 years in addition to the drugs of premier option for the remediation of migrant, perennial allergic rhinitis along with urticarial.

**SCHEME 1:**
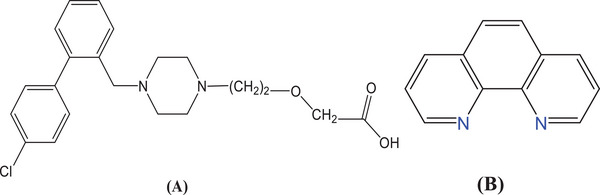
Structure of (A) (*RS*)‐2‐[2‐[4‐[(4‐chlorophenyl) phenyl methyl]piperazine‐l‐yl]‐ethoxy]acetic acid, dihydrochloride (CETZ.2HCl) and (B) 1,10‐phenanthroline monohydrate (Phen.H_2_O).

Over the last 20 years, newer medications have been released with a narrower range of negative effects. Measuring a drugs capacity to prevent a histamine‐induced cutaneous reaction is the most straightforward technique to assess the efficacy of histamine blockage at H1 receptor. Simons et al. [10] revealed the results of the first extensive comparison of first‐ in addition to second‐generation antihistamines, and they also noticed that the potencies were in the following order: placebo, astemizole, terfenadine, loratadine, cétirizine, and terfenadine [11]. The main metabolite of hydroxyzine, cetirizine (CETZ), has gained popularity as a treatment due to its great efficacy, low metabolism, and low occurrence of side effects [12, 13]. It treats a variety of allergy illnesses effectively as a second‐generation histamine H1 antagonist [11]. CETZ.2HCl (Scheme 1A) interacts with metal ions through the carboxylic acid moiety as a monodentate ligand in the deprotonated mode [14, 15, 16, 17, 18].

Phen.H_2_O (Scheme 1B) is a potent ligand that successfully donates nitrogen atoms to metal ions, resulting in the generation of stable chelates and providing additional characteristics for chelates because it possesses hetero aromatic in addition to aromatic groups [2, 19].

Many crucial biological processes involve metal chelation, where coordination can take place among a wide range of ions and a variety of ligands [3, 4]. Mixed‐ligand chelates consisting of bioactive ligands containing nitrogen or oxygen donor binding sites and M(II) ions were employed for biological, analytical, agricultural, industrial, and pharmaceutical purposes [5, 19]. Metal ions, such as copper and zinc, are vital for the health of humans and higher animals. These first‐row transition metals are associated with several proteins that play a crucial role in major physiological processes [20]. Many biological activities require copper, which is a vital trace element [21]. Copper's bio‐essential effectiveness along with oxidative easiness have encouraged many inorganic chemists to seek out copper(II)–CETZ chelates for distinct biological effectiveness, involving antibacterial, anticancer, antifungal, antioxidant, along with anti‐inflammatory features [22, 23, 24, 25, 26, 27, 28, 29]. Recently, the action of some drugs which coordinated with bio metals such as Cd(II), Zn(II), and Cu(II) utilized to treat metal‐dependent infections [30, 31]. Several reports were implicated in the histamine signaling pathway through “histamine H1‐receptor” in lipid and carbohydrate metabolism as well as associated diseases, including obesity and atherosclerosis [32, 33, 34]. On this basis, some researchers have proposed the H1 antihistamines drugs, such as azelastine, CETZ.2HCl, and fexofenadine, as anticipated adipogenic drugs [35, 36, 37]. However, their exact effects are yet disputed. Specifically, Raveendran et al. [34] proved that the stimulatory influence of CETZ.2HCl (H1 antihistamines) on the activity of lipase enzyme in high‐fat diet (HFD)‐treated mice; lipoprotein lipase (L. lipase) is an enzyme that performs a crucial role in the breakdown of circulating lipoproteins that are rich in triglycerides (TGs), particularly very low‐density lipoproteins (LDLs) and chylomicrons [38, 39]. The study conducted by Kobayashi et al. [38] showed a correlation between decreased L. lipase activity and a decline in plasma TGs as well as decreased levels of high‐density lipoprotein (HDL), commonly referred to as good cholesterol. These variables are thought to be risk factors in the development of atherosclerosis and coronary heart disease. The target of this investigation was to explore the impact of various biological elements, which include Cd(II), Zn(II), and Cu(II) on the efficacy of CETZ.2HCl in the existence of Phen.H_2_O. These distinctive neutral mononuclear metal chelates were synthesized and characterized utilizing spectroscopic techniques like UV–vis, x‐ray diffraction (XRD), ^1^H NMR, FT‐IR, elemental analyses (CHN), magnetic‐susceptibility studies, molar conductivity, and TG/DTG, differential thermal analysis (DTA). To determine the precise structure of the chelates and to calculate the total energy, heat of formation, along with total dipole moment, density functional theory (DFT) was applied. Moreover, we investigate a possible hypolipidemic of CETZ.2HCl and its metal complexes on the activity of lipoprotein lipase enzyme and lipid metabolism in C57BL/6 mice kept on HFDs. Thus, the present article opens the door to study pharmacological properties of such metal‐CETZ complexes on serum levels of lipid profiles, comprising TGs, LDL, total cholesterol (T. chol.), and good cholesterols like HDL. We are attempting to elucidate whether we are in a position to develop novel drugs that are likely to be used to treat obesity, atherosclerosis, or cardiovascular diseases in the future. Additionally, we assess the antimicrobial effectiveness of such compounds regarding some distinct bacteria in addition to fungal species.

## 2 Materials and Procedures

### 2.1 Solvents and Reagents

Analytical chemical reagents (AR), CETZ.2HCl (99%), Phen.H_2_O (99.5%), potassium hydroxide (99.9%), zinc chloride (99.9%), cadmium acetate dihydrate (99.7%), copper nitrate trihydrate (99.4%), silver nitrate (99.5%), ferric chloride (99.5%), ferrous sulfate (99%), potassium chromate (99%), absolute ethyl alcohol (99%), DMF (99%), and DMSO (99%) were acquired from DBK Pharmaceuticals, Aldrich plus Fluka Chemical Co. Every vessel had been submerged in a chromatic solution (potassium dichromate + conc. sulfuric acid) prior to getting carefully rinsed with bi‐distilled water as well as dehydrated in an oven at 100°C.

### 2.2 Computational Methods

Using the GAUSSIAN 98W package of programs [40], density functional theory at the B3LYP/CEP‐31G level of theory has been employed for computing the geometric parameters on geometries that were Cep‐31G basis set optimized. B3LYP is a key‐phrase for the hybrid functional that combines the Becke and Lee gradient functional in a linear way [41, 42, 43, 44], concurrent with the Hartree–Fock exchange function, Yang plus Parr [45].

### 2.3 Metal Chelates Preparation

The chelate green solid [Cu(CETZ)(Phen)(H_2_O)_2_(NO_3_)] (**1**) was synthesized through the addition of (1 mmol, 0.461 g) of CETZ.2HCl with (2 mmol, 0.336 g) of potassium hydroxide plus (**1** mmol, 0.0198 g) of Phen.H_2_O in 50 mL of EtOH. Following 10 min of agitation, 1 mmol of copper(II) nitrate trihydrate (0.241 g) in 20 mL EtOH was introduced to the mix and refluxed for 6 h. A green precipitate was obtained by slow evaporation for 7 days and dried over anhydrous CaCl_2_ under vacuum. White complexes [Zn(CETZ)(Phen)(H_2_O)_2_Cl] (**2**) and [Cd(CETZ)(Phen)(H_2_O)_2_(CH_3_COO)] (**3**) were made in the same manner that was earlier stated, using ethanol as the solvent, zinc chloride, and cadmium acetate dihydrate, respectively, in the proportions of 1:2:1:1 (CETZ.2HCl: KOH: Phen.H_2_O: M) molar ratio.

### 2.4 Instruments

FT‐IR spectra in KBr discs were obtained utilizing an FT‐IR 460 PLUS Spectrophotometer at 4000–400 cm^−1^. The ^1^H NMR spectra were generated using the Varian Mercury VX‐300 NMR spectrometer and DMSO‐d6 as the solvent. UV–vis spectra in DMSO were produced by UV‐3101PC Shimadzu. TGA‐50H Shimadzu utilized TG‐DTG measurements to analyze samples in a nitrogen (N_2_) environment, covering a temperature range from room temperature to 1000°C. Additionally, the sample mass was carefully quantified using an aluminum crucible. Atomic absorption, complexometric titration as well as thermogravimetry have employed to ascertain M percent content [30, 46, 47]. The entire metal content at the correspondent wavelength was estimated utilizing the direct approach of atomic absorption analysis. Different reference standard solutions for every metal were maintained at a certain concentration. A sufficiently illuminated Pye Unicam atomic‐absorption SP‐1900 spectrometer was utilized. The analyses have attained utilizing a Perkin Elmer 2400 CHN elemental analyzer. In order to assess melting points, a Buchi apparatus was used. Mercury tetrathiocyanatocobalt(II) was used as calibrant, and the magnetic moment of chelates was evaluated using a Sherwood scientific magnetic scale and a Gouy balance. CONSORT K410 has utilized to appreciate the molar conductance of 1 × 10^−3^ M solutions of compounds in DMF. XRD investigation was carried out using PW 1840 diffractometer, along with radiation supplied by a Cu anode 2000 W with an x‐ray tube operating at 40 kV in addition to 25 mA. All tests were performed at room temperature utilizing newly prepared solutions.

### 2.5 Animals and Treatment

This study employed 8‐week‐old male C57BL/6 mice of matched weight (around 20–25 g) from an inbred “genetically identical” strain. The mice were purchased from the National Research Center's Animal House. Then, they were subdivided into five groups, each group containing eight mice; four groups were treated with CETZ.2HCl (antihistamine drug) and its metal complexes, whereas one group was left untreated and classified as a control group. Animals were housed in modular plastic veterinary cages at air‐conditioned room, under pathogen‐free conditions [48, 49]. Mice were fed HFD, which consisted of carbs 42.0%, fat 23.0%, protein 17.0%, fiber 3.0%, minerals 5.0%, and moisture 10.0% for 3 months [50], together with administration of either CETZ.2HCl or its metal complexes (4 mg kg**^−1^** body weight) in DMF [37]. Experiments were performed in accordance with the regulations of the Animal Care and Use Committee of the College of Veterinary Medicine, Mansoura University (MU‐ACUC), Egypt; MU‐ACUC (PHARM.R.24.02.30), complied with “Guide for the Care and Use of Laboratory Animals” (National Research Council publication 8th ed., USA, 2011) [51].

### 2.6 Biochemical Assays

Specimens of blood were gathered from the retro‐orbital sinus, under halothane (1.5 percent v/v halothane in oxygen) anesthesia via inhalation. The collected blood was allowed to clot, by centrifugation at 1600 rpm for 20 min, to obtain serum. Isolated sera were maintained at −80°C until tests of liver function in addition to lipid profiles were performed. For estimation of L. lipase activity, fluorometric L. lipase activity assay kit was utilized following the manufacturer's protocol (Cat. No. STA‐610, Cell BIOLABS INC., San Diego, USA). Serum T. chol. LDL, HDL, TG, ALT (alanine aminotransferase), AST (aspartate aminotransferase), and ALP (alkaline phosphatase) were examined with Roche kits using COBAS Integra 400 and COBAS Integra‐400 plus (Roche Diagnostics Limited, Switzerland).

### 2.7 Antimicrobial Investigation

A modified Beecher alongside Wong method was used to assess the antibacterial effect of CETZ.2HCl, Phen.H_2_O, and their chelates toward numerous isolates of bacteria, as instance *Bacillus subtilis*, *Salmonella typhi, Staphylococcus aureus*, and *Escherichia coli*, as well as against all phytopathogenic fungi, *Aspergillus terreus* and *Aspergillus niger* [52]. Both antibacterial and antifungal nutrition agar mediums were made, cooled to 37°C, along with seeded with the studied microbial species. The antibacterial nutrient agar medium consisted of 1.5% agar, 0.5% peptone, 0.5% sodium chloride, 0.2% yeast extract, and then 0.1% beef extract [53]. After the material had solidified, a sterile cork borer has utilized to make 5 mm diameter holes following dissolving the investigated compounds in DMF at 10^−3^ M and placing them in petri‐dishes. Each of the experiment plates had been incubated for 20 h at 37°C for bacteria and 7 days at 30°C for fungi. The same methodology has revealed the antimicrobial property of the standard (ampicillin, gentamicin, and clotrimazole). A minimum inhibitory concentration for CETZ.2HCl, Phen.H_2_O, and their metal chelates was examined toward bacterial and fungal strains (MIC) as well as the minimum bactericidal concentration (MBC), the lowest concentration of an antibacterial agent required to kill a bacterium over a fixed, somewhat extended period, such as 18 or 24 h, was measured [54]. Concentrations of all compounds varied between 0.025 and 0.1 g mL**^−1^**. All compounds’ activity index percent was calculated employing the following equation by dividing the zone of inhibition (ZOI) of the test compound by the corresponding standard:

(1) $$\begin{array}{ccc} & & \%\;\mathrm{Activity}\;\mathrm{index}\\ & & =\frac{\mathrm{Zone}\;\mathrm{of}\;\mathrm{inhibition}\;\mathrm{by}\;\mathrm{test}\;\mathrm{compound}\;\left(\mathrm{diametre}\right)}{\mathrm{Zone}\;\mathrm{of}\;\mathrm{inhibition}\;\mathrm{by}\;\mathrm{standard}\;\left(\mathrm{diametre}\right)}\times 100. \end{array}$$

### 2.8 Molecular Docking

The docking process was performed adopting the reported procedures [55, 56] and was visualized using Discovery Studio Visualizer 2021 [57]. Predicting the binding interactions of newest synthesized CETZ chelates was the goal of these investigations; Cu(II) and Zn(II) chelates the most potent complexes, then Cd(II) complex. The results were compared to those of CETZ.2HCl, or the parent drug in complexes containing Phen and selected metals. The tested complexes were docked against lipoprotein L. lipase (ID: 6E7K) [58].

## 3 Results and Discussion

### 3.1 Elemental and Molar Conductivity

The structures and properties of the newly synthesized CETZ mixed‐ligand metal complexes were characterized, and Table 1 summarizes outcomes of chelates physical properties alongside the elemental analysis. According to the results of the elemental investigations of our complexes, 1:1:1 (CETZ.2HCl:Phen.H_2_O:metal) stoichiometry was supported. All chelates’ molar conductance measurements with 1 × 10^−3^ M DMF varied between 4.91 and 11.53 Ω^−1^ cm^2^ mol^−1^; such low values demonstrated the chelates’ non‐electrolytic nature [59, 60]. Qualitative data confirmed the occurrence of nitrate, acetate, and chloride across the complex sphere.

**TABLE 1:** Physico‐analytical data along with elemental analysis for CETZ.2HCl, Phen.H_2_O along with their chelates.

Compounds M.Wt (M.F.)	Yield% (Mp °C^−1^)	Color	Found (Calc.) %					*Λ* Ω^−1^ cm^2^ mol^−1^
			C	H	N	M	Cl	
(CETZ.2HCl) 461.80 (C_21_H_27_N_2_O_3_Cl_3_)	— (112)	White	54.46 (54.57)	5.78 (5.84)	6.00 (6.06)	—	22.93 (23.03)	22.81
(Phen.H_2_O) 198.20 (C_12_H_10_N_2_O)	—(100)	White	72.53 (72.65)	4.98 (5.04)	14.08 (14.12)	—	—	5.00
(**1**) 728.99 (CuC_33_H_36_N_5_O_8_Cl)	78.44 360	Green	53.01 (54.32)	4.98 (4.94)	9.51 (9.60)	8.60 (8.72)	4.79 (4.86)	11.53
(**2**) 704.28 (ZnC_33_H_36_N_4_O_5_Cl_2_)	83.91 (>360)	White	56.01 (56.23)	5.18 (5.11)	7.88 (7.95)	9.21 (9.28)	9.98 (10.07)	8.29
(**3**) 774.86 (CdC_35_H_39_N_4_O_7_Cl)	86.88 (>360)	White	54.00 (54.20)	5.09 (5.03)	7.17 (7.23)	14.39 (14.51)	4.48 (4.58)	4.91

### 3.2 FT‐IR Spectra in Addition to Mode of Bonding

**SCHEME 2:**
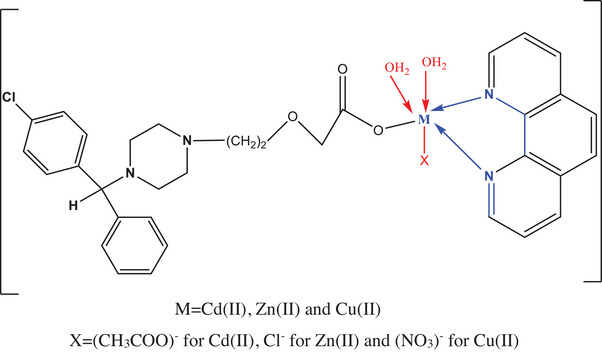
The coordination mode of Cetz.2HCl, Phen.H_2_O with Cd(II), Zn(II), and Cu(II) ion.

FT‐IR of CETZ.2HCl, Phen.H_2_O along with their metal chelates, is scheduled in Table 2 in addition to depicted in Figure S1. All vibrational peaks due to *ν*_as_(COO^−^), *ν*_s_(COO^−^), in addition to *ν*(C═N), were assigned. FT‐IR spectrum of CETZ.2HCl showed *v*(O─H) in addition to *v*(COOH) of carboxylic group at 3433 and 1735 cm^−1^ [61, 62]. A comparison between the spectra of CETZ.2HCl and the respective chelates discloses the vanishing of absorption band at 1735 cm^−1^ pointing out the chelation of CETZ.2HCl with metal ions [62]. Moreover, new ones that develop in the area 1604–1620 cm^−1^ could be correlated to asymmetric *ν*_as_(COO^−^) and 1388–1419 cm^−1^ for symmetric *ν*_s_(COO^−^) stretching vibration with Δ*ν* = *ν*_as_(COO^–^) − *ν*_s_(COO^−^) values falling in an area higher than 200 cm^−1^ for our chelates, providing evidence of a monodentate coordination mode of carboxylate group [63, 64, 65, 66]. The absorption band at 1586 cm^−1^ that correlates to v(C═N) in the spectrum of Phen.H_2_O in addition to the alteration of this band to a lower frequency (around 1575 cm^−1^) in all chelates provide evidence that the pyridine nitrogen atoms of Phen.H_2_O chelate to the metal ions in a bidentate manner [67, 68, 69]. All complexes’ spectra showed prominent bands at around 3435, 841, and 605 cm^−1^, which were correlated to the coordinated water molecule's *v*(O–H) rocking and wagging motions [70, 71]. Cu(II) complex displayed some bands associated to *v*(N═O) (*ν*_5_), *v*_as_(NO_2_) (*ν*_1_), and *ν*_s_(NO_2_) (*ν*_2_) at 1419, 1311, and 1030 cm^−1^, respectively, supporting the coordinated nitrate's with metal ions [72, 73]. The distinction between mono, bidentate chelating nitrates, and bridging mode has been made using the separation Δ*ν* = ν_5_ − ν_1_, and the value of Δ*ν*(108 cm^−1^) indicates a monodentate nitrate. The emergence of additional peaks with varying intensities *v*(M–O) as well as *v*(M–N) found at 640 and 486 w cm^−1^ for copper(II), at 640 and 478 w cm^−1^ for zinc(II), and at 632 and 524 w cm^−1^ for Cd(II) was evidence that the oxygen and nitrogen atoms of CETZ.2HCl and Phen.H_2_O were indeed coordinated (Scheme 2) [66].

**TABLE 2:** Preferred FT‐IR bands along with assignments of CETZ.2HCl, Phen.H_2_O beside their chelates.

Compounds	*ν*(O–H); H_2_O; COOH	*ν*(C=O); COOH	*ν*_as_(COO^−^)	*ν*(C=N) in pyridyl	*ν*_s_(COO^−^)	∆*ν*	*ν*(NO_3_)^−^			∆*ν*	*ν*(M–O) and *ν*(M–N)
							*ν*_5_	*ν_2_*	*ν_1_*		
CETZ.2HCl	3433mbr	1735vs	—	—	—	—	—	—	—	—	—
Phen.H_2_O	3380mbr	—	—	1586m	—	—	—	—	—	—	—
(**1**)	3441mbr	—	1604vw	1577w	1388w	216	1419m	*1311m*	*1030m*	*108*	640w, 486w
(**2**)	3433sbr	—	1618m	1576m	1415s	203	—	—	—	—	640w, 478w
(**3**)	3433sbr	—	1620m	1575m	1419s	201	—	—	—	—	632m, 524w

Abbreviations: br, broad; m, medium; s, strong; w, weak; *ν*, stretching.

### 3.3 Magnetic Moment Measurements in Addition to Electronic Spectra

Electronic spectroscopy is powerful method that can be used to explain the chelation process and the molecular structure of the chelates. Table 3 lists the absorption spectra of CETZ.2HCl, Phen.H_2_O, and their chelates in DMSO at wavelengths ranging from 200 to 800 nm. CETZ.2HCl showed three peaks, potentially corresponding to π–π* and n–π* at 257, 264, and 335 nm (Figure S2) [73, 74]. Moreover, peaks at 273 and 310, 350 nm in the spectrum of Phen.H_2_O may be designed to π–π* and n–π* transitions [75, 76]. The occurrence of additional peaks for chelates in 452–554 nm regions could be designated to ligand–metal charge transfer (LMCT) [77]. UV–vis spectrum of Cu(II) chelate revealed absorption band at 16 366 cm^−1^ that linked to d–d transition (^2^B1g→^2^E1g) with crystal field stabilization energy (CFSE) equal −117 +4P with value of 10 Dq at 196 kJ mol^−1^. Cu(II) chelate has a measured magnetic moment of 1.70 B.M., which suggests that the complex is octahedral geometry [78, 79]. The molar absorptivity (*ε*) of the synthesized chelates was calculated utilizing Lambert–Beer's law equation: *A* = *ε* cl (2), where *A* = absorbance, *c* = 1.0 × 10^−3^ M, l = length of cell (1 cm).

**TABLE 3:** Ultraviolet–visible spectra of CETZ.2HCl, Phen.H_2_O, along with their chelates.

Compounds	Peak		Assignment	ε*(M^−1^ cm^−1^) x 10^4^	10 Dq		CFSE
	nm	cm^−1^			cm^−1^	kJ mol^−1^	
CETZ.2HCl	257	38 910	π→π*	0.166	—	—	
	264	37 878	π→π*	0.652			
	335	29 850	n→π*	0.804			
Phen.H_2_O	273	41 152	π→π*	1.50			
	310	36 630	n→π*	2.00			
	350	28 571	n→π*	0.400			
**(1)**	248	40 322	π→π*	1.00	16 366	196	−117 +4P
	267	37 453	π→π*	1.29			
	297	33 670	n→π*	0.500			
	554	18 050	LMCT	0.100			
	611	16 366	^2^B1g→^2^E1g	0.090			
**(2)**	267	37 453	π→π*	1.48			
	301	33 222	n→π*	0.452			
	327	30 581	n→π*	0.120			
	513	19 493	LMCT	0.089			
**(3)**	267	37 453	π→π*	2.65			
	293	34 129	π→π*	0.901			
	325	30 769	n→π*	0.102			
	452	22 123	LMCT	0.098			
	508	19 685	LMCT	0.087			

### 3.4 ^1^H NMR Spectroscopy

Proton NMR spectra of CETZ.2HCl, Phen.H_2_O along with their chelates, were done, and the data are illustrated in Figure S3 in addition to summarized in Table 4. New signals appeared in the chelates spectra in the region of 3.40–3.63 ppm might be ascribed to existence of water molecules [80, 81]. According to the coordination from the carboxylate oxygen atom, the singlet at 11.4 ppm caused by the carboxylic acid proton noticed in the spectrum of CETZ.2HCl is not seen in the complexes spectra, which is proportionate with the datum formerly acquired from the infrared spectra and molar conductivity [82, 83]. A small shift for δH, –CH aromatic peaks of CETZ.2HCl and Phen.H_2_O (7.30–7.79) and (7.12–8.70 ppm) was compared with their peaks of complexes (7.12–9.11 ppm). In the spectra of the complexes, it can be seen that all peaks of the free ligands were existing with a chemical shift as a result of complexation [84, 85, 86].

**TABLE 4:** ^1^H NMR data (ppm) as well as preliminary assignments for CETZ.2HCl, Phen.H_2_O, and their chelates.

CETZ.2HCl	Phen.H_2_O	(1)	(2)	(3)	Assignments
3.19	—	2.51	3.09	2.48	δH,–CH in piperazine ring
3.38	—	3.04	3.27	3.17	δH, –CH in NCH_2_
3.64	—	3.22	3.51	3.31	δH, –CH in CH_2_O
—	—	3.41	3.63	3.40	δH, H_2_O
4.08	—	3.65	3.77	3.49	δH, –CH in CH_2_COOH
5.21	—	5.20	4.38	4.13	δH, –CH in methyldiphenyl group
7.30–7.79	7.12–8.70	7.12–8.21	7.99–8.89	8.02–9.11	δH, –CH aromatic
11.4	—	—	—	—	δH, –COOH

### 3.5 TG and DTG

The findings of the compound weight losses are presented in Table 5 and illustrated in Figure S4. Thermal study outcomes of the complexes were consistent with the molecular formula established by elemental analysis results. TG of CETZ.2HCl commenced at 165°C and concluded at 400°C, exhibiting two distinct stages. The initial stage takes place at *T*_max_ 186°C, resulting in a weight loss of 7.79% (calculated as 7.89%) due to the release of HCl. The second stage, which occurs at *T*_max_ 286°C and results in a mass loss of 92.21% (Calc. as 92.11%), is attributed to the decomposition of 10C_2_H_2_ + CO + N_2_ + 2H_2_O + 2HCl. This stage is characterized by activation energy (*E*_a_) 50.23 kJ mol^−1^ with reaction order of 0.983. TG curve of Phen.H_2_O was discussed in literature [87].

**TABLE 5:** Weight loss values and maximum temperature *T*_max_ (°C) for CETZ.2HCl, Phen.H_2_O, and their chelates.

Compounds	Decay	*T*_max_ (°C)	Weight loss (%)		Lost species
			Calc.	Found	
CETZ.2HCl (C_21_H_27_N_2_O_3_Cl_3_)	Step 1 Step 2 Total loss Residue	186 286	7.89 92.11 100.0 —	7.79 91.37 99.16 —	HCl 2HCl + 10C_2_H_2_ + 2H_2_O + CO + N_2_
Phen.H_2_O (C_12_H_10_N_2_O)	Step 1 Step 2 Total loss Residue	95 278	9.08 90.92 100 —	8.98 90.87 99.85 —	H_2_O 2C_4_H_2_ + 2C_2_H_2_ + N_2_
**(1)** (CuC_33_H_37_N_5_O_9_Cl)	Step 1 Total loss	344 662	82.96 82.96	83.02 83.02	13C_2_H_2_ + 3NH_3_ + HCl + 3CO_2_ + 2NO
	Residue		17.04	16.98	CuO + 4C
**(2)** (ZnC_33_H_37_N_4_O_5_Cl_2_)	Step 1 Total loss	456, 521	83.78 83.78	83.70 83.70	15C_2_H_2_ + 2HCl + 3NO + NH_3_ + H_2_O
	Residue		16.22	16.30	ZnO + 3C
**(3)** (CdC_35_H_40_N_4_O_7_Cl)	Step 1 Total loss	479, 659	80.36 80.36	80.20 80.20	16C_2_H_2_ + 2NH_3_ + CO + 0.5Cl_2_ + H_2_O + 2NO_2_
	Residue		19.64	19.80	CdO + 2C

TG curves of complexes **(1)**, **(2),** and **(3)** exhibit one degradation step, occurred at two maxima (344, 662), (456, 521), and (479, 659)°C associated with elimination of 13C_2_H_2_ + 3NH_3_ + HCl + 3CO_2_ + 2NO, 15C_2_H_2_ + 2HCl + 3NO + NH_3_ + H_2_O and 16C_2_H_2_ + 2NH_3_ + CO + 0.5Cl_2_ + H_2_O + 2NO_2_ giving CuO + 4C, ZnO + 3C and CdO + 2C as final products, respectively. Thermal residue of chelates had distinguished by infrared spectra as displayed in Figure S5.

Utilizing Coats–Redfern [88] along with Horowitz–Metzger [89] methods, activation energy (*E*_a_) of the decay phases has calculated from TG along with DTG thermograms to be able to evaluate the influence of the complexes’ structural features on thermal behavior. Figure S6 and Table 6 illustrate that *E*_a_ of decomposition ranged within the range of 50.23–194.41 kJ mol^−1^. Elevated levels of *E*_a_ demonstrate the chelates’ thermal stability [90, 91]. The negative values for ΔS* show that the reactions are occurring more slowly than they would normally [92]. Moreover, the processes of breakdown are endothermic because Δ*H* has positive values.

**TABLE 6:** Kinetic parameters for CETZ.2HCl, Phen.H_2_O, and their chelates.

				Parameter					*R* ^a^	SD^b^
Compounds	Decomposition range (K)	*T*_s_ (K)	Method	*E*_a_ (kJ mol^−1^)	*A* (s^−1^)	Δ*S* ^*^ (kJ mol^−1^ K^−1^)	Δ*H* ^*^ (kJ mol^−1^)	ΔG* (kJ mol^−1^)		
CETZ.2HCl (C_21_H_27_N_2_O_3_Cl_3_)	471–1066	559	CR HM	50.23 52.76	1.10 × 10^2^ 2.87 × 10^2^	−0.2110 −0.2030	45.58 47.78	168.20 161.29	0.983 0.970	0.187 0.250
Phen.H_2_O (C_12_H_10_N_2_O)	394–572	551	CR HM	117.83 146.78	2.03 × 10^9^ 7.97 × 10^11^	−0.0718 −0.0222	113.25 142.20	152.84 154.42	0.996 0.998	0.120 0.076
**(1)** (CuC_33_H_39_N_5_O_9_Cl)	570–791	617	CR HM	78.22 77.13	3.81 × 10^3^ 1.37 × 10^4^	−0.1823 −0.1717	73.09 72.00	185.63 177.97	0.974 0.964	0.198 0.232
**(2)** (ZnC_33_H_39_N_4_O_6_Cl_2_)	625–760	729	CR HM	117.63 145.10	1.60 × 10^6^ 1.36 × 10^8^	−0.1335 −0.0963	111.57 139.03	208.93 209.48	0.974 0.968	0.181 0.201
**(3)** (CdC_35_H_42_N_4_O_8_Cl)	533–763	752	CR HM	105.44 194.41	3.83 × 10^6^ 2.19 × 10^11^	−0.1265 −0.0354	99.19 188.15	194.36 214.84	0.980 0.976	0.151 0.167

^a^ Correlation coefficients of the Arrhenius plots.

^b^ Standard deviation.

### 3.6 Differential Thermal Analysis

**FIGURE 1:**
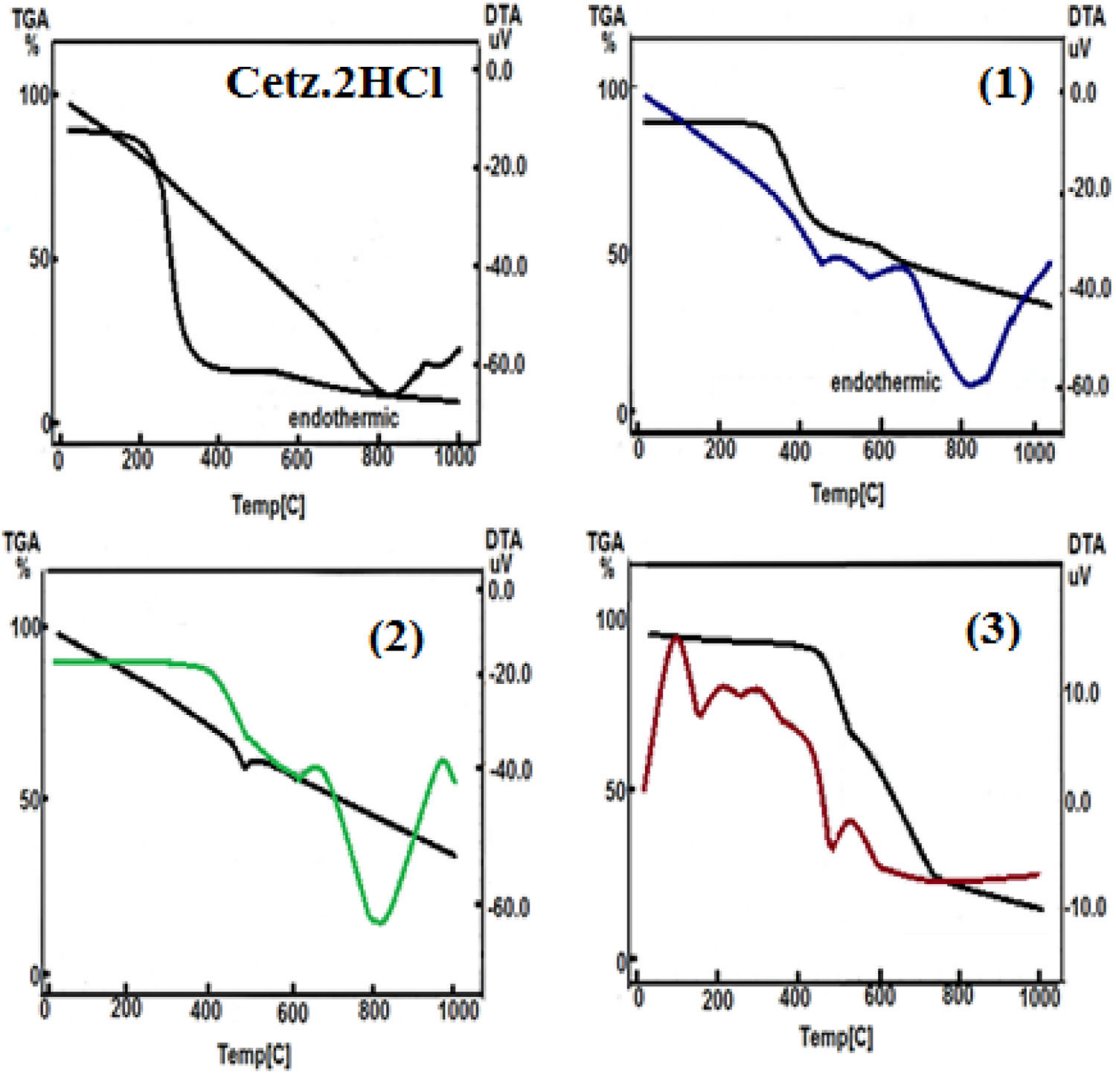
DTA diagrams for CETZ.2HCl and its complexes. DTA, differential thermal analysis.

DTA thermogram of CETZ.2HCl in addition to its metal chelates under research displays many phases, in accordance with the prior DTA assessment, as illustrated in Figure 1. The chemical changes that occur after the elimination of water, anion, in addition to ligand molecules, were shown in the DTA curves in addition to identified as exo‐ or endothermic peaks. At 810°C, the CETZ.2HCl offers one peak as endothermic at −14.98 uv. The complex (**1**) manifested three endothermic peaks at −1.42, −0.59, and −18.97 uv with values of 449°C, 456°C, and 819°C, respectively. Diverse endothermic peaks at 484°C, 596°C, and 825°C with activation energies of −5.77, −2.77, and −50.33 uv for complex (**2**). The complex (**3**) has two endothermic peaks at −4.41 and −3.02 uv with *T*_max_ 146°C and 474°C.

### 3.7 X‐Ray Diffraction

**FIGURE 2:**
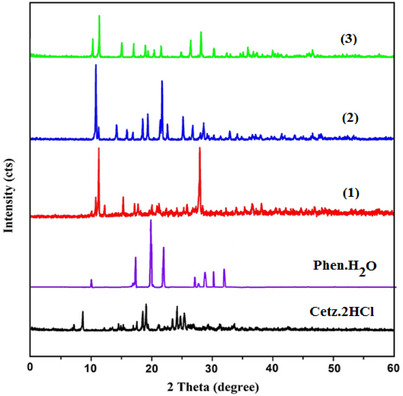
XRD spectra for CETZ.2HCl, Phen.H_2_O, and their metal chelates.

In the utilization of several procedures, like crystallization through gradual evaporation in addition to cooling, we were not capable of acquiring adequate monocrystals to conduct x‐ray crystallographic observations. In the absence of x‐ray crystallographic study, we enhance technical quality of the manuscript by x‐ray powder diffraction (Figure 2 and Table 7). The diffraction of compounds with the greatest intensity (100%) appears at 2*θ* = 19.08, 19.87, 11.36, 10.89, and 11.45 for CETZ.2HCl, Phen.H_2_O, **(1)**, **(2)** in addition to **(3)**, respectively. The crystallite sizes of the investigated substances were estimated utilizing formula (3) developed by Debye–Scherer.

**TABLE 7:** An average size of the crystallite for CETZ.2HCl, Phen.H_2_O, and their chelates assessed from XRD form.

Compounds	2*θ* (°)	*d* value (A°)	Relative intensity (%)	Full width at half maximum (FWHM)	Average crystallite size (*d*), (nm)	Dislocation density (*δ*) × 10^−3^ (nm^−2^)
CETZ.2HCl	19.08	4.65	100	0.0984	81.86	0.149
Phen.H_2_O	19.87	4.47	100	0.217	37.16	0.724
(**1**)	11.36	7.79	100	0.08	97.71	0.105
(**2**)	10.89	8.12	100	0.09	81.09	0.152
(**3**)	11.45	7.73	100	0.08	97.72	0.105

The crystallite sizes of the produced chelates were discovered to be among 81.09–97.72 nm that belongs to the diverse range of nanoscale structures (Table 7):

(2) $$d=\frac{k\;\cdot \lambda }{\beta \;\cdot \;\cos\theta }$$

where *λ* is the wavelength of the x‐ray beam (0.15405 nm), *d* is the mean particle diameter, assuming spherical particles, *β* is the full width at half maximum (FWHM) of the diffracted peak in (radians), and *k* is the Scherer constant (=0.9) along with *θ*, which is the angle of diffraction (radians). The chelates’ dislocation density (*δ*) was in the 0.105–0.152 × 10^−3^ nm^−2^ range, where δ is the number of dislocation lines per unit area of the crystal via the following equation [93]:

(3)

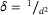




### 3.8 Antimicrobial Efficiency for the Compounds

The antimicrobial impacts of CETZ.2HCl, Phen.H_2_O, along with their metal chelates toward featured bacterial as well as fungal species, were examined via disc diffusion methodology (Figure S7) [94, 95, 96, 97]. As depicted in Table 8, the biological activity results, in terms of the ZOI, indicate that complex **(1)** exhibited a highly significant effect against *E. coli* and *S. typhi*, as well as a considerable effect against *S. aureus* and *B. subtilis*. Complex **(2)** demonstrated a substantial degree of significance in comparison to *B. subtilis* and *S. typhi*, whereas complex **(3)** revealed an exceptionally high level of significance against *B. subtilis*, followed by significance against *E. coli* and *S. typhi*. A comparison of Cu(II) chelate within the chelates, as well as past attempts, is presented in Table 9 and Figure S8. CETZ exhibited the lowest MIC and MBC (µg mL**^−1^**) values for *S. aureus* at concentrations of 0.025 and 0.049, followed by chelate (**2**) at 0.050 and 0.057, and subsequently followed by complex (1) at 0.075 and 0.086. The lowest MIC and MBC for *B. subtilis*, on the other hand, were 0.025 and 0.032 for Phen.H2O, complexes (1) and (**2**), CETZ.2HCl, and complex (**3**). Additionally, complex (**2**) had the lowest MIC and MBC for *E. coli* at 0.050 and 0.062, followed by complex (**3**) and CETZ.2HCl. Regarding the antifungal activities, the lowest MIC value was shown for *S. typhi* when treated by CETZ.2HCl at value 0.25 followed by complexes (**2**) and (**3**) at 0.050 then accompanied by complex (**1**) at 0.075. The lowest MIC for *A. niger* was demonstrated by CETZ.2HCl and complex (**2**) at 0.025 followed by complex (1) at 0.050. Furthermore, MIC with the lowest value for *A. terreus* was displayed for CETZ.2HCl at 0.025 then complex (**2**) at 0.050 followed by complexes (**1**) at 0.075 and finally at 0.100 at complex (**3**) (Figure S9).The lipid membrane that covers the cell and the permeability of the cell allow lipid‐soluble chemicals to enter the cell more easily, which is a crucial factor in determining the effectiveness of an antibacterial agent. It implies that chelation may promote metal‐CETZ complexes’ transit beyond the lipid layer of the cell membrane to the region of action [98, 99]. The activity index for all compounds has measured (Figure S10 **)**.

**TABLE 8:** Inhibitory diameter zone (ZOI, mm), minimum inhibitory concentration (MIC, µg mL**^−1^**), minimum bactericidal concentration (MBC, µg mL**^−1^**) along with the activity index (%) measurements for CETZ.2HCl, Phen.H_2_O, and their metal chelates.

Compounds								Tested *G*(+) and *G*(−)							
	*Staphylococcus aureus*				*Bacillus subtilis*			*Escherichia coli*				*Salmonella typhi*			
	ZOI (mm)	AI (%)	MIC (µg mL^−1^)	MBC (µg mL^−1^)	ZOI (mm)	AI (%)	MIC (µg mL^−1^)	ZOI (mm)	AI (%)	MIC (µg mL^−1^)	MBC (µg mL^−1^)	ZOI (mm)	AI (%)	MIC (µg mL^−1^)	MBC (µg mL^−1^)
CETZ.2HCl	9.4 ± 0.02	41.4	0.025 ± 0.02	0.049 ± 0.01	12.1 ± 0.15	42.6	0.050 5±0.00	8.5 ±0.50	43.6	0.075 ± 0.01	0.091 0.014±	7.7 ±0.58	37.2	0.025 ± 0.03	0.037 ± 0.01
Phen.H_2_O	^NA^	—	—	—	1.1 ± 0.16	3.87	0.025 ±0.03	NA	—	—		NA	—	—	
**(1)**	17.3 ^+^ ^2^ ±0.33	76.2	0.075 ±0.03	0.086 ±0.004	23.3 ^+^ ^2^ ± 0.66	82.04	0.050 ±0.007	32.5 ^+^ ^3^ ±0.30	166.6	0.1 ±0.006	0.15 0.02±	30.0 ^+^ ^3^ ±0.10	144.9	0.075 ±0.006	0.081 ± 0.01
**(2)**	15.2 ^+^ ^1^ ±0.74	66.9	0.050 ±0.007	0.057 ±0.002	21.2 ^+^ ^2^ ±1.04	74.65	0.050 ±0.01	7.9^NS^ ±0.34	40.5	0.050 ±0.02	0.063 ± 0.015	18.0 ^+^ ^2^ ±0.17	86.9	0.050 ±0.007	0.065 ± 0.014
**(3)**	NA	—	—	—	32.0 ^+^ ^3^ ±0.68	112.68	0.075 ±0.006	28.5 ^+^ ^2^ ±0.83	146.1	0.1 ±0.02	0.23 ± 0.053	19.5 ^+^ ^2^ ±0.85	94.2	0.050 ±0.01	0.066 ± 0.014
Standard	22.7	—	—	—	28.4	—		19.5	—	—	—	20.7	—	—	

Compounds	Tested Fungal Strains					
	*Aspergillus niger*			*Aspergillus terreus*		
	ZOI (mm)	AI (%)	MIC (µg mL^−1^)	ZOI (mm)	AI (%)	MIC (µg mL^−1^)
CETZ.2HCl	12.5 ±0.36	107.8	0.025 ±0.01	10.3 ±0.27	58.9	0.025 ±0.01
Phen.H_2_O	NA	—	—	NA	—	—
**(1)**	13.1^NS^ ±0.38	66.16	0.050 ±0.02	10.5^NS^ ±1.06	60.0	0.075 ±0.005
**(2)**	17.6 ^+^ ^1^ ±0.43	88.88	0.025 ±0.01	12.0^NS^ ±1.71	68.57	0.050 ±0.02
**(3)**	NA	—	—	14.7 ^+^ ^1^ ±0.55	84.0	0.1 ±0.01
Standard	19.8	—		17.5	—	

Abbreviations: IQR, interquartile range; SD, standard deviation.

*Note*: Qualitative data were analyzed using the chi‐square test or Fisher's exact test (median (*N* value) ± IQR), whereas quantitative (normally distributed) data were expressed in (mean (*N* value) ± SD) and were compared using Student's *t*‐test. As compared to controls, the *p* < 0.05 considered statistically highly significant, *P* ^NS^ *p* not significant, *p* > 0.05 considered *p* significant, *p* < 0.01 *p* very highly significant. Standard: ampicillin, gentamicin, and colitrimazole for *G* ^+^, *G* ^−^, and fungi species, respectively. Statistical significance *P* ^NS^ *p* not significant, *p* > 0.05; *P* ^+1^ *p* significant, *p* < 0.05; *P* ^+2^ *p* highly significant, *p* < 0.01; *P* ^+3^ *p* very highly significant, *p* < 0.001;Student'*s t*‐test (paired).

**TABLE 9:** Comparison of Cu(II)–cetirizine (CETZ) complex in the chelates versus preceding research.

Complexes	Microbial species											
	Bacteria								Fungi			
	*Staphylococcus aureus*	AI	*Bacillus subtillis*	AI	*Escherichia coli*	AI	*Salmonella typhi*	AI	*Aspergillus niger*	AI	*Aspergillus terreus*	AI
[(Cu(CETZ)(Bipy)(H_2_O)_2_(NO_3_)]^[^ ^68^ ^]^	24 ^+^ ^3^ ± 0.13	105.7	15.7 ^+^ ^1^ ± 0.001	55.3	22.1 ^+^ ^2^ ± 0.005	113.3	12.4 ^+^ ^1^ ± 0.22	59.9	17.6 ^+^ ^1^ ± 0.11	88.89	NA	
[Cu(CETZ)(phen)(H_2_O)_2_(NO_3_)]	17.3 ^+^ ^2^ ± 0.33	105.7	23.3 ^+^ ^2^ ± 0.66	55.3	32.5 ^+^ ^3^ ± 0.30	113.3	30.0 ^+^ ^3^ ± 0.10	59.9	13.1^NS^ ± 0.38	88.89	NA	—
[Cu(AMBI)(CETZ)(NO_3_)(H_2_O)_2_]^[^ ^11^ ^]^	26.5 ± 0.6	96.72	28.9 ± 0.3	89.2	21.3 ± 0.4	95.10	—	—	—	—	—	—
[Cu(CETZ)(Ala)(H_2_O)]^[^ ^7^ ^]^	15.6 ± 0.4	56.93	13.9 ± 0.3	42.9	11.8 ± 0.5	52.91	—	—	—	—	—	—
[Cu(CETZ)_2_(H_2_O)_2_].H_2_O^[^ ^9^ ^]^	15.21 ± 0.46	—	18.31 ± 0.55	—	13.43 ± 0.39	—	—	—	—	—	—	—

Abbreviations: IQR, interquartile range; NA, no activity; SD, standard deviation.

*Note*: Qualitative data were analyzed using the chi‐square test or Fisher's exact test (median (*N* value) ± IQR), whereas quantitative (normally distributed) data were expressed in (mean (*N* value) ± SD) and were compared using Student *t*‐test. As compared to controls, the *p* < 0.05 considered statistically highly significant, *P* ^NS^ *p* not significant, *p* > 0.05 considered P significant, *p* < 0.01 *p* very highly significan. Standard: ampicillin, gentamicin, and colitrimazole for *G* ^+^, *G* ^−^, and fungi species, respectively. Statistical significance *P* ^NS^ *p* not significant, *p* > 0.05; *P* ^+1^ *p* significant, *p* < 0.05; *P* ^+2^ *p* highly significant, *p* < 0.01; *P* ^+3^ *p* very highly significant, *p* < 0.001; Student'*s t*‐test (paired).

### 3.9 The Hypolipidemic Effect of Synthetic Compounds

**FIGURE 3:**
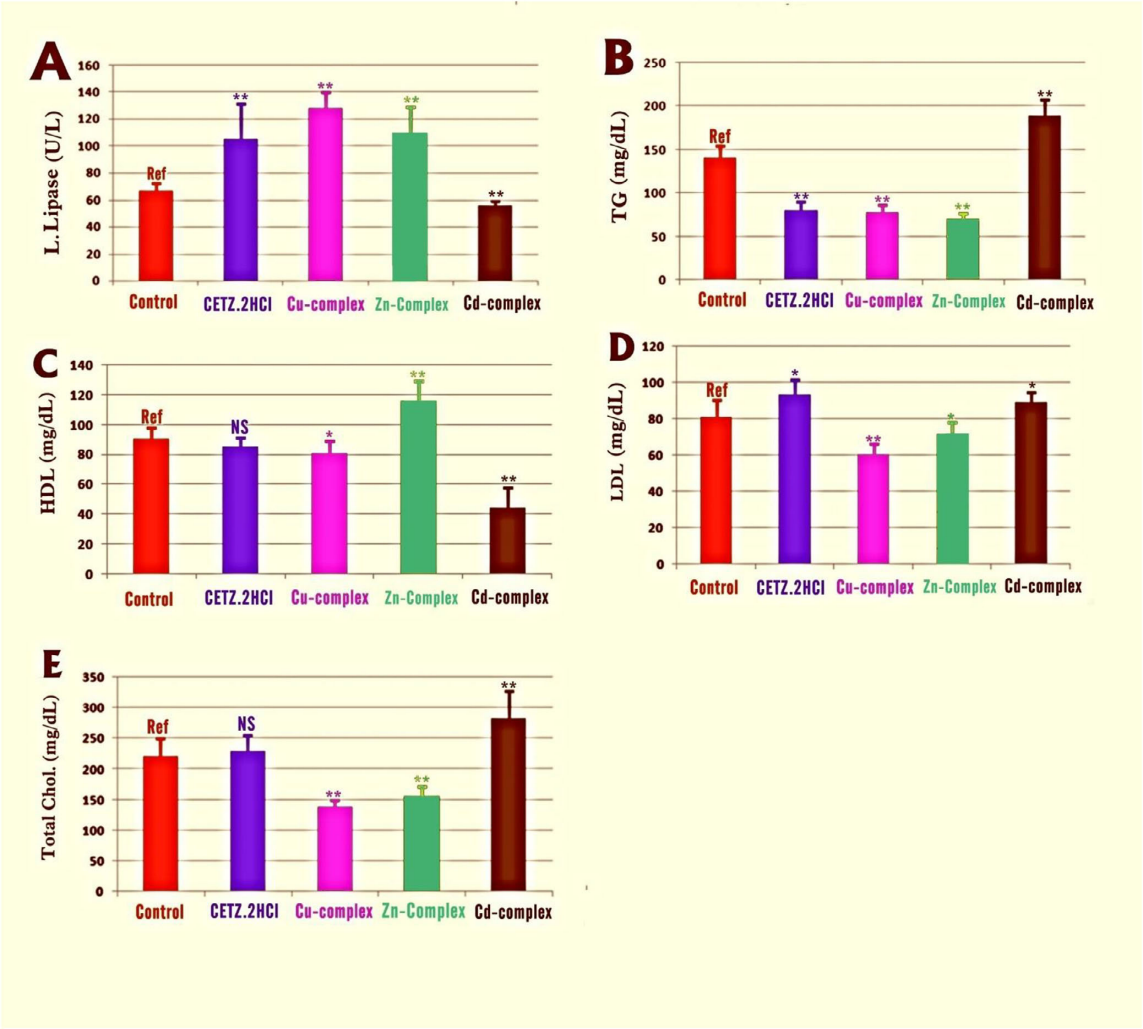
Effect of CETZ.2HCl antihistamine drug along with its chelates on the serum levels of lipase enzyme and lipid profile of high‐fat diet mice. Serum levels of lipase (A), triglycerides (B), high‐density lipoprotein (C), low‐density lipoprotein (D), and total cholesterol (E) were evaluated. Student's *t*‐test analyses were performed between drug‐treated groups versus controls, considering differences as ^**^highly significant (*p < 0.01*), ^*^significant (*p < 0.05*), and nonsignificant (NS*, p > 0.05*). HDL, high‐density lipoprotein; LDL, low‐density lipoprotein; TG, triglyceride.

As shown in Figure 3 and Table S1, compared with controls, levels of L. lipase were significantly elevated in mice treated with CETZ.2HCl and its metal complexes, but not in Cd(II) complex‐treated, which had the reverse effect. Moreover, hence, these synthetic antihistamines successfully enhanced lipase enzyme activity. This was confirmed by the TGs serum levels, which were significantly reduced in mice treated with CETZ.2HCl, Cu(II) and Zn(II) chelates, coinciding with the induction of L. lipase enzyme activity. It is worth noting that serum levels of T. chol., LDL “bad cholesterol,” were increased, concurrently with a decrease in HDL “good cholesterol” levels in animals treated with Cd‐CETZ complex and CETZ.2HCl alone, but not significant. However, Cu(II) and Zn(II) chelates direct the lipid metabolism in a way that lowers blood levels of TG, T. chol., LDL “bad cholesterol,” and raises HDL “good cholesterol,” thus having a pronounced hypolipidemic impact.

### 3.10 Liver Toxicity of Synthetic Complexes

As shown in Table 10, all liver function enzymes revealed a tendency toward an increment in sera of mice subjected to CETZ.2HCl and its chelates, with the exception of the Zn(II) chelate, which succeeded in alleviating the drug‐induced liver toxicity evidenced by a decrease in ALT, AST as well as ALP serum levels compared to controls.

**TABLE 10:** Comparing the serum liver enzyme levels of mice given CETZ.2HCl antihistamine and its metal complexes to those of animals not given the medication.

Animal groups	Liver function enzymes (mean ± SD)		
	ALT (U L^−1^)	AST (U L^−1^)	ALP (U L^−1^)
Untreated (controls)[ref.]	181.88 ± 13.18	280 ± 36.03	61.88 ± 6.38
Treated with CETZ.2HCl	245.63 ± 43.99^*^	345.5 ± 57.73^*^	90.5 ± 16.79^**^
Treated with Cu(II) complex	228.63 ± 50.57^*^	274.63 ± 36.40^NS^	88 ± 12.64^**^
Treated with Zn(II) complex	164.87 ± 35.27^**^	156.5 ± 16.12^**^	49.63 ± 6.16^**^
Treated with Cd(II) complex	283.75 ± 23.49^**^	374.88 ± 36.33^**^	123.63 ± 11.45^**^

Abbreviations: IQR, interquartile range, SD, standard deviation.

*Note*: Qualitative data were analyzed using the chi‐square test or Fisher's exact test (median (*N* value) ± IQR), whereas quantitative (normally distributed) data were expressed in (mean (*N* value) ± SD) and were compared using Student's *t*‐test. As compared to controls, the *p* < 0.05 considered statistically significant, *p > *0.05 considered non significant, *p* < 0.01 *p* highly significant. Student's *t*‐test analyses were performed between drug‐treated groups versus controls, considering differences as ^**^highly significant (*p* < *0.01*), ^*^significant (*p* < *0.05*), and nonsignificant (*NS, p > 0.05*).

### 3.11 Molecular Docking and Molecular Dynamics Simulation Against L. Lipase Active Site

**FIGURE 4:**
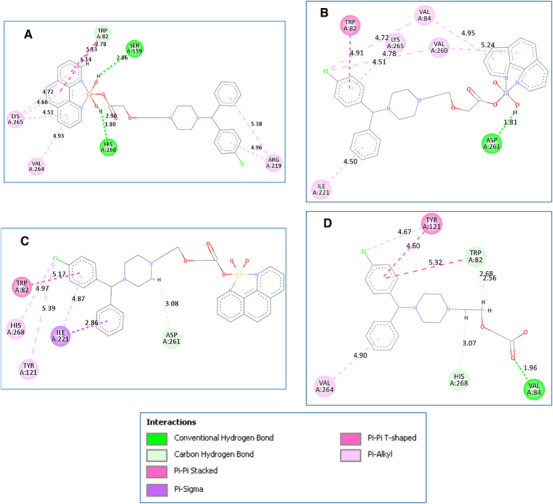
2D binding mode and residues involved in the recognition of (A) Cu(II), (B) Zn(II), (C) Cd(II) chelates, and (D) CETZ docked and minimized in the SOD binding pocket.

Figure 4, Figure S11, and Table S2 reveal the results of molecular docking for experimentally synthesized CETZ chelates. Results indicated that metal complexes containing Cu(II) and Zn(II) chelates, with the exception of Cd(II), exhibited the higher binding affinities to the active site of L. lipase compared to CETZ alone (with mean L. lipase activity = 104.9 U L**^−1^,** binding score = −9.6 kcal mol**^−1^**), because CETZ is bound to L. lipase active site by its terminal carboxylic group via hydrogen bond with Val84 amino acid residue, in addition to other weak hydrophilic interactions with Trp82 and His268 by its side chain. Moreover, it is also bound to L. lipase pocket by its phenyl rings via π–π stacking interactions with Trp82 and Tyr121 amino acid residues. Upon analyzing the metal complexes data, it was found that Cu(II) and Zn(II) chelates experienced the highest levels of target binding affinities and docking scores (with mean L. lipase activity of 127.9 and 108.9 U L**^−1^** and binding scores of −12.4 and −10.8 kcal mol**^−1^**). Cu(II) complex shared CETZ in binding to His268, Trp82, and Val264 amino acid residues. Furthermore, Cu(II) complex is bound to L. lipase active site by its highly polar central metal‐complex core via hydrogen bond with Ser159 and His268 amino acid residue, and other weak hydrophilic interactions with Trp82 by its Phen moiety. Zn(II) complex shared CETZ in binding to Trp82 amino acid residue via π–π interactions. Additionally, Zn(II) complex is bound to L. lipase by its highly polar central metal‐CETZ complex core via hydrogen bond with Asp261 amino acid residue, and other π‐alkyl interactions with Val84, Ile221, Val260, and Lys265 by its phenyl rings and Phen moiety.

On the other hand, Cd(II) complex (mean L. lipase activity = 52.4 U L**^−1^,** binding score = −5.5 kcal mol**^−1^**) differs from other complexes in that it is exposed out of the L. lipase active site with weak binding affinity to target that was shown clearly in its 3D pose in Figure S11.

### 3.12 ADMET Prediction Study

The evaluated metal–CETZ complexes ADME‐toxicity pharmacokinetic properties were anticipated utilizing Swiss ADME and pre‐ADMET predictors [100, 101], and data are reported in Table 11. These complexes can be readily absorbed from the gastrointestinal tract (GIT), with a remarkably high human intestinal absorption percentage (HIA%) of around 98%. Their water solubility is relatively low to moderate, with computed log *S* values ranging from −7.6 to −7.9. Toxicity simulation outcomes revealed that all of the tested chelates are non‐inhibitors of the CYP1A2 and CYP2C9 enzymes, essential members of liver cytochrome P450 metabolic enzymes, indicating that they have low probability to cause drug–drug interactions. Furthermore, hERG (human‐ether‐a‐go‐go‐related gene) channel inhibition test that is enrolled in cardiac repolarization showed that they have medium risk to inhibit hERG channel causing QT prolongation.

**TABLE 11:** Predicted ADMET data for the newly synthesized chelates.

Complexes	HIA%	Log *S*	CYP1A2 inhibitor	CYP2C9 inhibitor	hERG_inhibition
Cu(II) complex	98.03	−7.6	Non‐inhibitor	Non‐inhibitor	Medium_risk
Zn(II) complex	98.04	−7.6	Non‐inhibitor	Non‐inhibitor	Medium_risk
Cd(II) complex	98.07	−7.9	Non‐inhibitor	Non‐inhibitor	Medium_risk

### 3.13 Structural Parameters in Addition to Model of CETZ.2HCl

**SCHEME 3:**
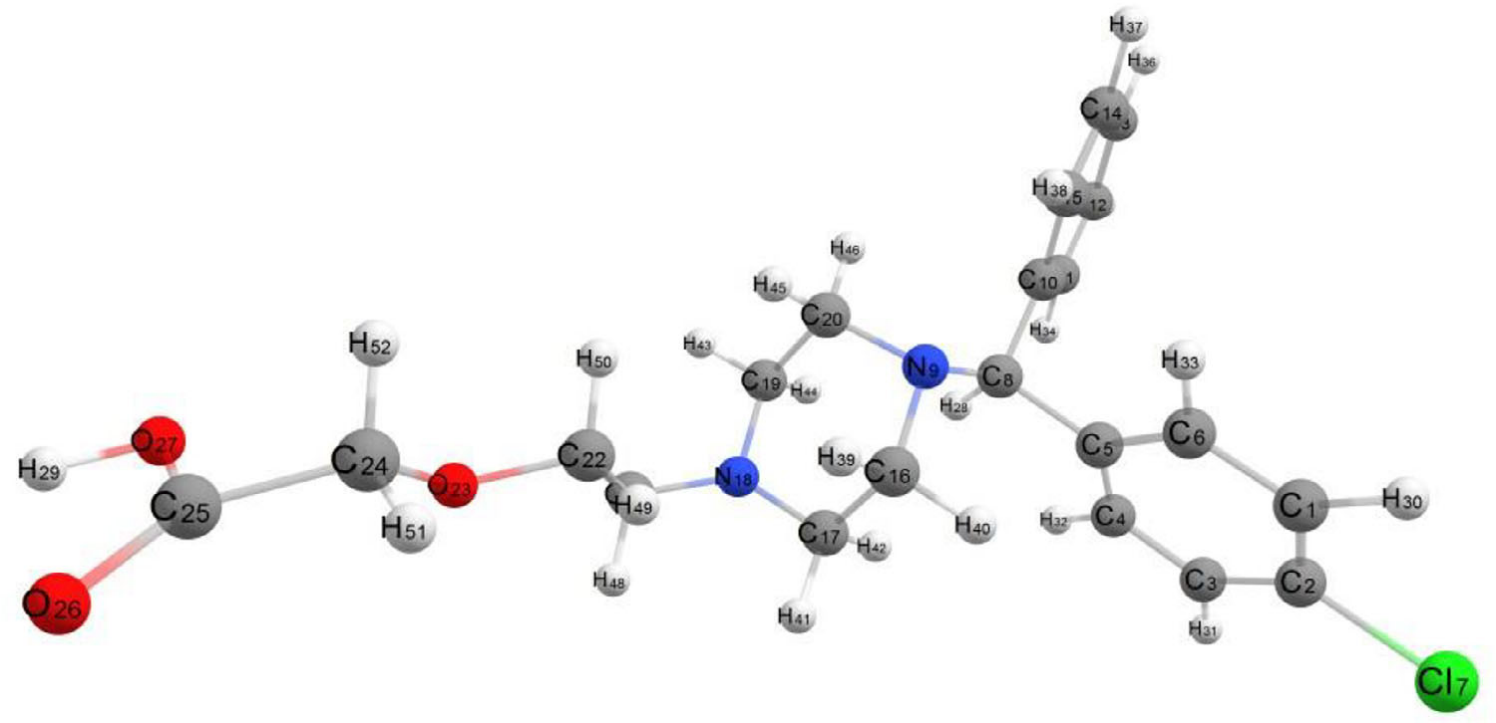
Optimial geometry of CETZ.2HCl utilizing DFT methodology at B3LYP level with CEP‐31G as bases set.

Geometric equilibrium parameters for CETZ.2HCl were estimated using DFT calculations (Table S3) [37, 38]. Experimental data support that carboxylate group is coordinated as an ionic monodentate which agrees with DFT calculation data. According to dihedral angles, the carboxylate group is not found in the plane of ethoxy group which linked to the piperazine ring and found in the same plane of piperazine ring (Scheme 3). As compared to the x‐ray data, **CETZ's** optimized geometry is more convenient [37]. The bond lengths C25O26, C25O27, C16 N9, N9 C20, C17 N18, and C19 N18 are 1.206, 1.332, 1.453, 1.457, 1.448, and 1.449 Å, respectively. The calculated bond angles for CETZ.2HCl found in the range of 68.31°–126.56°. The calculated dipole moment and the total energy of CETZ.2HCl are 4.26 D and −15 813.849 kcal mol^−1^.

### 3.14 Geometrical Structure of Complexes

The empirical findings show that Cu(II), Zn(II) and Cd(II) interacted with CETZ.2HCl, Phen.H_2_O, along with two H_2_O molecules, to constitute the chelation sphere., NO_3_^−^, Cl^−^, and CH_3_COO^−^ producing deformed octahedral structures (Schemes S1–S3). The theoretical data support that the equatorial plane is occupied by N1, N4, O7 (H_2_O) in addition to O8 (NO_3_^−^) for complex (1), N1, N4, O5, and O7 for complex (**2**), N1, N4, O5, and O7 for complex **(3),** whereas the axial plane contains O5 and O6 for complex (1), O6 and Cl8 for complex **(2),** and O6 and O8 for complex **(3)**. The angle O6‐M‐O7 is 84.20°, 83.41°, and 100.13° for complexes **(1)–(3),** respectively (Table S4), which demonstrate that H_2_O molecules were positioned in *cis* with respect to one another. The energies of complexes (**1)–(3)** are −310 833.820, −249 442.317, and −270 148.039 kcal mol**^−1^** with higher dipole moment values of 24.619, 14.19, and 17.381 D, respectively.

### 3.15 Charge Distribution Analysis

On the basis of a natural population analysis, the charge distribution analysis for the optimal geometry configuration for our complexes was conducted (NPA) (Table S5). The data indicate that Cd(II) complex shows higher charge density with a charge accumulated on Cd(II) ion 0.326, whereas Cu(II) and Zn(II) chelates carry lower charge density of 0.008 and 0.120. Although N1 and N4 of Phen.H_2_O, and O5 of CETZ.2HCl have delocalized negative charges, all hydrogen atoms in all chelates have positive charges. The charge density beyond the O5 atom for CETZ.2HCl reduced from −0.379 in free state to −0.009‐(−0.488) range for the three complexes. The charge on N1 and N4 of Phen.H_2_O are reduced from −0.209 to −0.213 in free state to −0.043‐(−0.110) and −0.062‐(−0.126) range for N1 and N4, respectively. Carbons C2 and C3 of Phen.H_2_O that have a connection to the nitrogen atoms N1 and N4 exhibit greater positive values because nitrogen atoms are electronegative. These findings indicate an electron back‐donation to the CETZ.2HCl π* orbitals from metal positions in an MLCT style. Comparison of the estimated charge densities for the chelates, in addition to the donating atoms, O5 of CETZ.2HCl, confirmed this conclusion.

### 3.16 Frontier Molecular Orbitals

**FIGURE 5:**
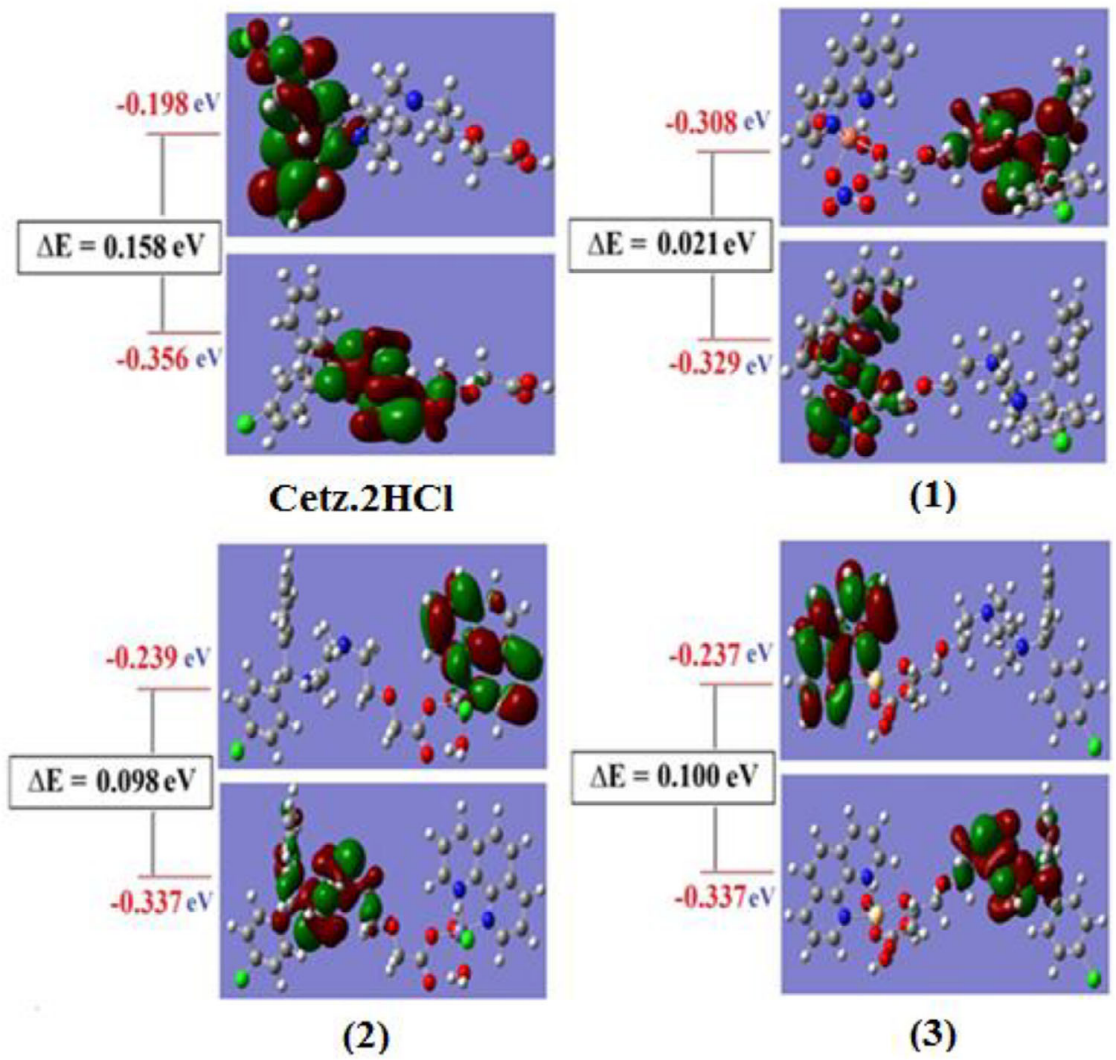
Energy levels along with molecular orbital surfaces of **CETZ.2HCl** in addition to its metal chelates using DFT calculations.

For the investigated chelates, the energy gap (Δ*E*) varied between 0.021 eV for the more reactive spin up orbitals Cu(II) chelate and 0.100 eV for the less reactive Cd(II) chelate, allowing for easy energy transfer between these orbitals, which is why we can see a peak for the complexes at 250 nm in their UV–vis spectra. Δ*E* for CETZ.2HCl and Phen.H_2_O are 0.158 and 0.243 eV. Figure 5 depicts that nodal features of the examined chelates’ molecular orbitals show that there are few nodal planes, substantial orbital overlap, in addition to orbital delocalization. The various MOs exhibit various levels of localization on the diverse complex fragments, and the previously stated rationale is true for MO analysis of all the examined chelates. All studied chelates have lower Δ*E* than CETZ.2HCl so these chelates are more reactive. The hardness (*η*) can be expressed as *η* = (*I* − *A*)/2 where *I* is the ionization energy, and *A* is the electron affinity. The softness of all chelates varied between 0.0105 for Cu(II) chelate and 0.050 for Cd(II) chelate, whereas *η* for CETZ.2HCl and Phen.H_2_O are 0.079 and 0.122. On the basis of these criteria, Cu(II) chelate is absolutely soft with *σ* = 95.238 eV; however, Cd(II) chelate is treated as hard chelate (*σ* = 20,000 eV). Regarding the free ligands, all investigated compounds were regarded as soft complexes, and the (σ) of CETZ.2HCl along with Phen.H_2_O was 12.658 in addition to 8.197 eV.

### 3.17 Excited State

The TD‐DFT at the B3LYP level of G03W programmer demonstrated which it provided a clear illustration of ultraviolet–visible spectra [102, 103, 104]. The calculation of the excitation energies was hybrid functionalized by Bauernschmitt alongside Ahlrichs [104]. These hybrid techniques are frequently noticeably better than conventional Hartree–Fock (HF)‐based techniques. With this piece, the wave functions of SCF MOs have thoroughly examined, and all calculations after that used the optimal geometry were determined. The findings indicate which various chemical orbitals exhibit some degree of electron delocalization. As demonstrated in Table S5, the Cu(II)–CETZ complex's HOMO exhibits delocalization of the electronic density, whereas its LUMO exhibits localization of the electron density by 100% on CETZ.2HCl with no portion on any other components of the complex. When it comes to Cd(II) and Zn(II) chelates, the electron density is entirely localized on CETZ.2HCl with 100% for HOMO, whereas in LUMO, the majority of the electron density is localized on Phen.H_2_O with 94.2% and 5.8% on chloride for Zn(II) but is 90.2% on Phen.H_2_O with a small portion on water molecule for Cd(II). Electronic transition can be defined as a combination of n→π* along with π→π* transitions. The agreement between the theoretical in addition to experimental sections demonstrates that the carboxylate group's oxygen atom, which CETZ.2HCl uses as a monodentate ligand, is what causes the reaction with the metal ion. When all transition states were compared to experimental data, they all supported one another.

## 4 Conclusions

Metal‐CETZ complexes are depicted by physicochemical in addition to spectroscopic methods. According to FT‐IR data, complexation process with Cd(II), Zn(II), and Cu(II) has demonstrated the chelation potential of oxygen atom for CETZ.2HCl along with nitrogen atoms for Phen.H_2_O. The data of magnetic moment and electronic spectra supported the chelation of CETZ.2HCl plus Phen.H_2_O with metal ions forming distorted octahedral geometry. TG analysis showed the chelates disintegrated with the metal oxide and carbon residue in a single step. Coats Redfern plus Horowitz–Metzger procedures were applied to assess each of the kinetic variables (*E*_a_, ΔS*, ΔH*, along with ΔG*). DFT calculations of the molecular modeling validate the molecular geometry and pointed out that all chelates were soft regarding with two ligands. In this study, CETZ.2HCl and its metal complexes, except Cd(II)complex, increased the activity of l‐lipase enzyme leading to an expected decrease in TG level in HFD mice. Moreover, we noted nonsignificant alterations in both T.chol. and HDL “good cholesterol” in mice treated with CETZ.2HCl alone. Despite this, Zn(II) complex enhances the hypolipidemic efficacy of the parent ligand, which is expressed as a significant decrease in bad cholesterols, such as T. chol. and LDL in line with a significant increase in HDL “good cholesterol.” Interestingly, Zn(II) complex has more evident hepatoprotective effect compared to other complexes and CETZ.2HCl alone. Inversely, Cd(II) complex delivered the worst effects due to exacerbated liver toxicity and hyperlipidemia. Regarding the antimicrobial efficacy, Zn(II) complex shows significant against *A. niger*, whereas Cd(II) complex shows significant against *A. terreus*. Cu‐CETZ complex shows highly significant activity toward *S. aureus* and *B. subtilis* in addition to very highly significant activity toward *E. coli* or *S. typhi* compared with other compounds. In the end, docking simulation tests demonstrated the enhanced affinity of newly developed Zn(II) and Cu(II) chelates to the L. lipase enzyme, causing enhanced activation of the enzyme. The predicated ADME‐Tox findings of new metal‐CETZ chelates demonstrated that they have better GIT absorption and lower drug–drug interaction in addition to a medium risk of cardiac toxicity probabilities.

## Author Contributions

**Sherif M. Abd El‐Hamid**: supervision, formal analysis, writing–original draft, writing–review and editing. **Sadeek A. Sadeek**: supervision, formal analysis, writing–original draft, writing–review and editing. **Ahmed E. Salem**: investigation, methodology. **Amira A. Mohamed**: investigation, methodology. **Soha F. Mohammed**: validation. **Hazem S. Elshafie**: Validation. **Wael A. Zordok**: visualization, writing–original draft, writing–review and editing. **Safa W. Aziz**: visualization, writing–original draft, writing–review and editing. **Mohamed A. Sabry**: formal analysis, molecular docking, writing. **Adriano Sofo**: formal analysis, molecular docking, writing. **Mohammed S. El‐Gedamy**: methodology; enzyme activity and lipid profile testing, writing original draft.

## Conflicts of Interest

The authors declare no conflicts of interest.

## Supporting information



Supporting Information

## Data Availability

Data will be made available on request.
