# Supplementary material for: Structural, Spectroscopic, and Docking Analysis of N,O‐Donor Ligand Metal Complex Nanoparticles With Hypolipidemic Effects via Lipoprotein Lipase Activation in High‐Fat Diet Mice
Source: Chem Biodivers. 2024 Dec 27;22(5):e202403003. doi: 10.1002/cbdv.202403003 (PMC12081029; doi:10.1002/cbdv.202403003)
Supplement: Supplementary file 1 — Supporting Information [file CBDV-22-e202403003-s001.pdf]

Supplementary Materials

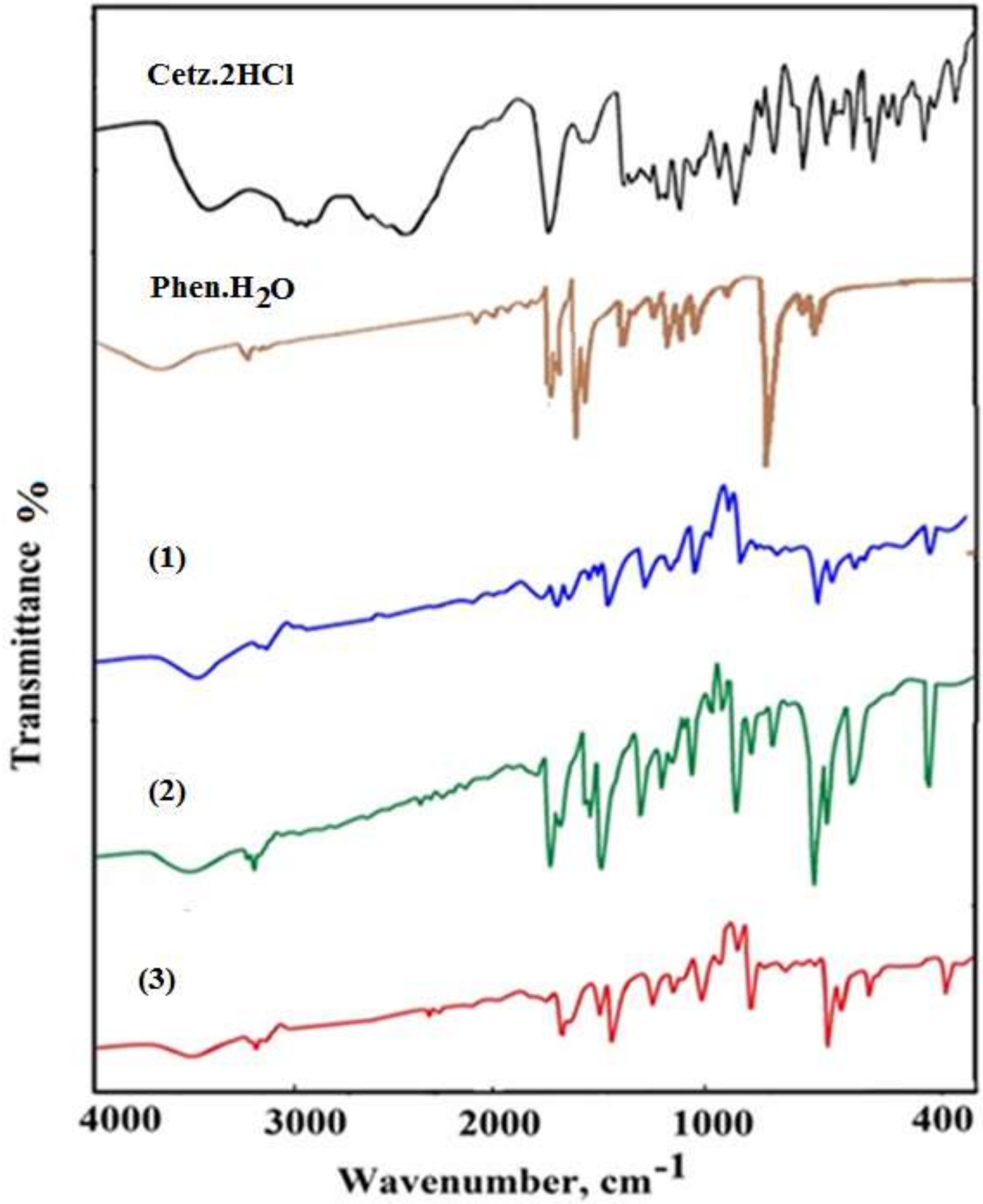

Figure S1. Infrared spectra for CETZ.2HCl, Phen.H_2O and their metal complexes.

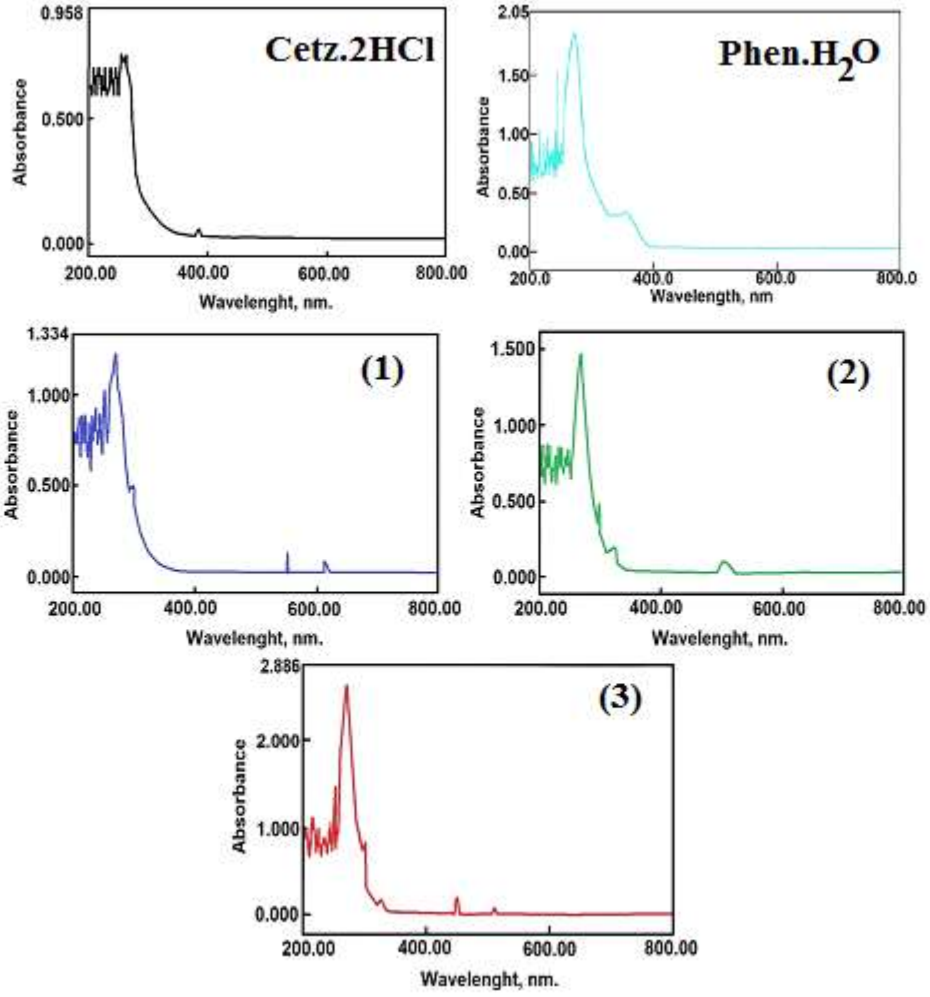

Figure S2: Electronic absorption spectra for CETZ.2HCl, Phen.H_2O and their metal complexes.

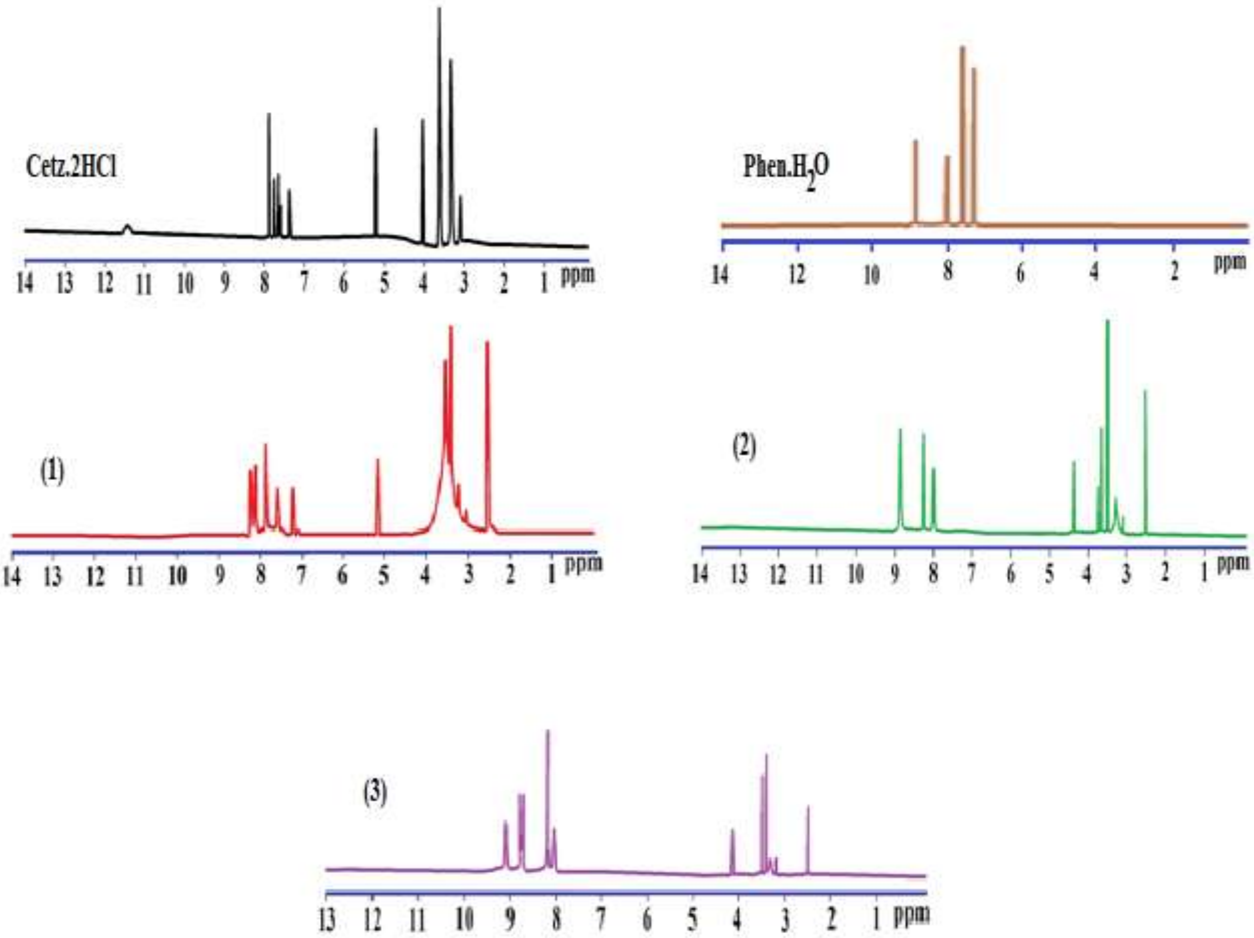

Figure S3: ^{1}H NMR spectra for CETZ.2HCl, Phen.H_2O and their metal complexes.

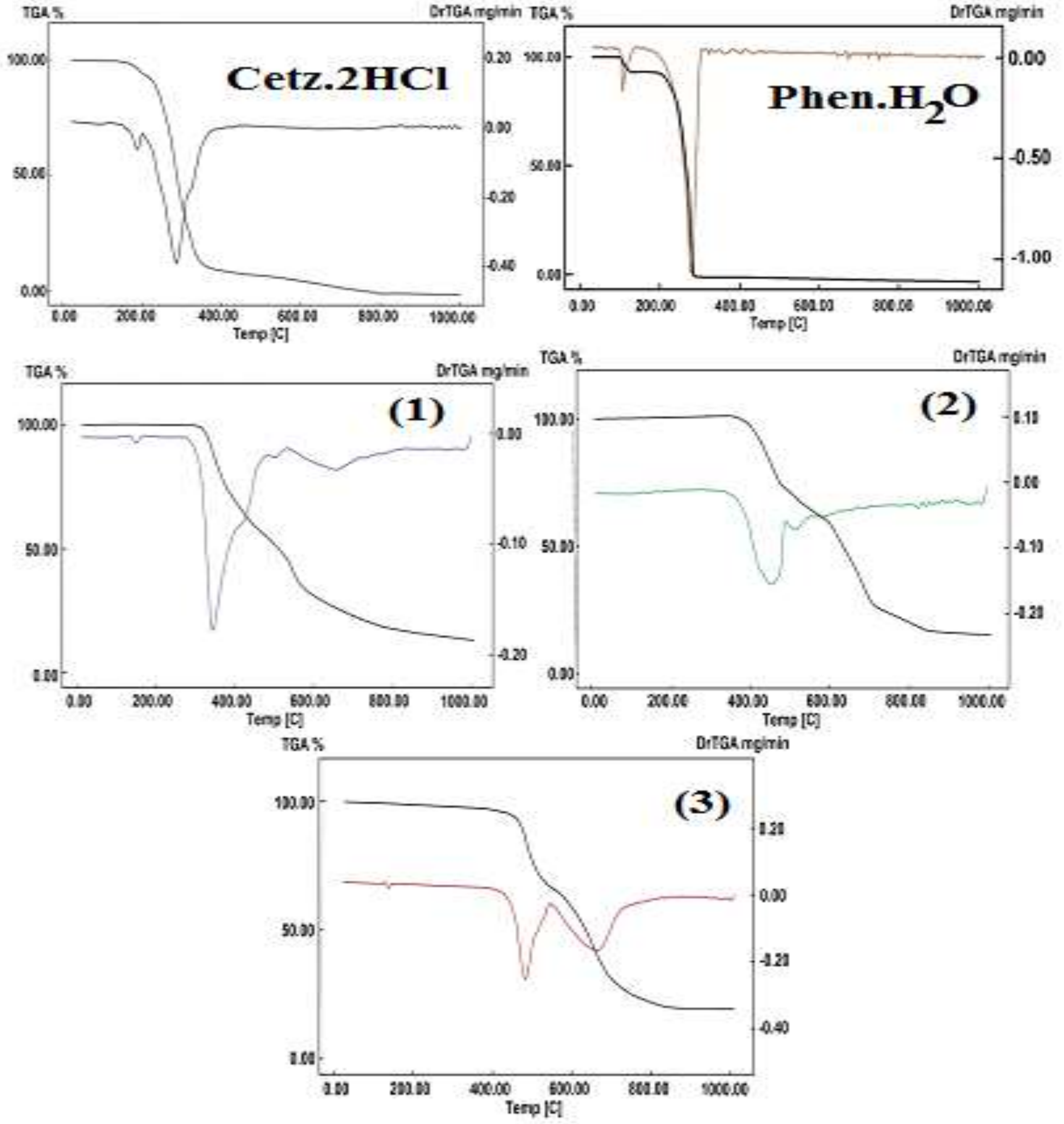

Figure S4: TG and DTG diagrams for CETZ.2HCl, Phen.H_2O and their metal complexes.

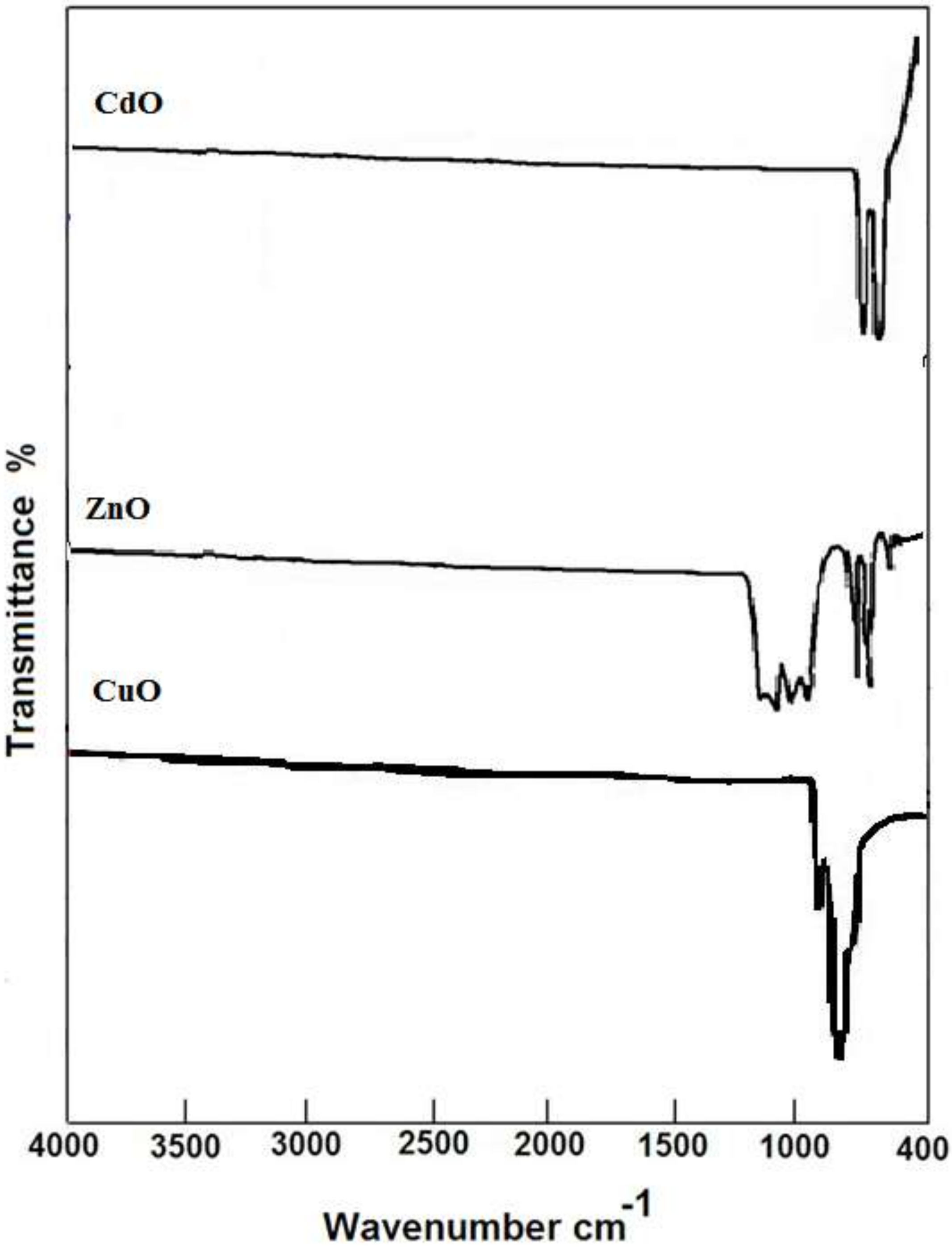

Figure S5: IR spectra for CuO, ZnO and CdO.

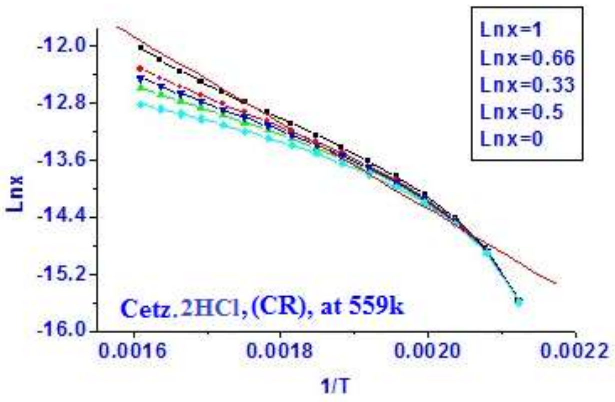
Lnx=1
Lnx=0.66
Lnx=0.33
Lnx=0.5
Lnx=0
Lnx
1/T
Cetz.2HCl, (CR), at 559k

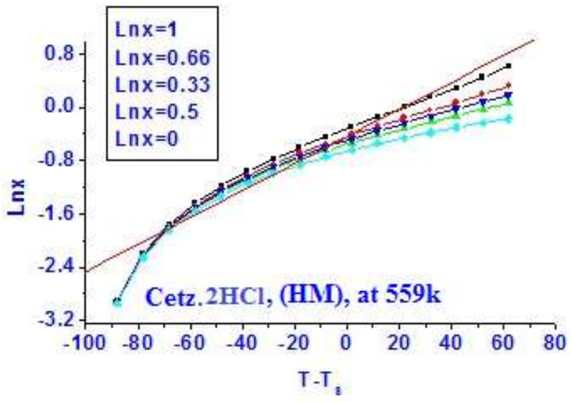
Lnx=1
Lnx=0.66
Lnx=0.33
Lnx=0.5
Lnx=0
Lnx
T-Tg
Cetz.2HCl, (HM), at 559k

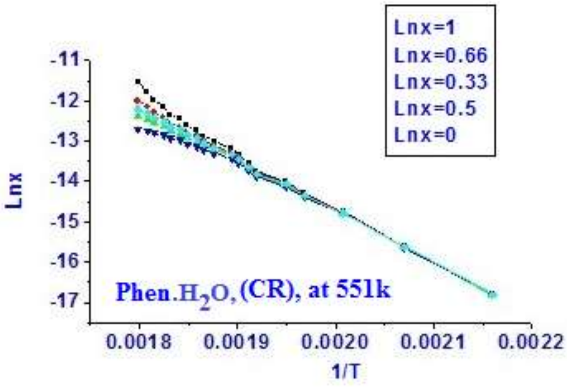
Lnx=1
Lnx=0.66
Lnx=0.33
Lnx=0.5
Lnx=0
Lnx
1/T
Phen.H2O, (CR), at 551k

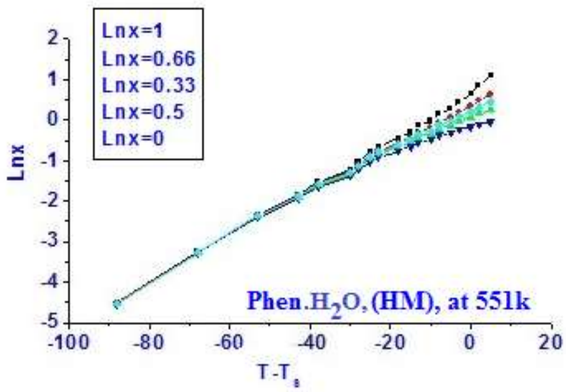
Lnx=1
Lnx=0.66
Lnx=0.33
Lnx=0.5
Lnx=0
Lnx
T-Tg
Phen.H2O, (HM), at 551k

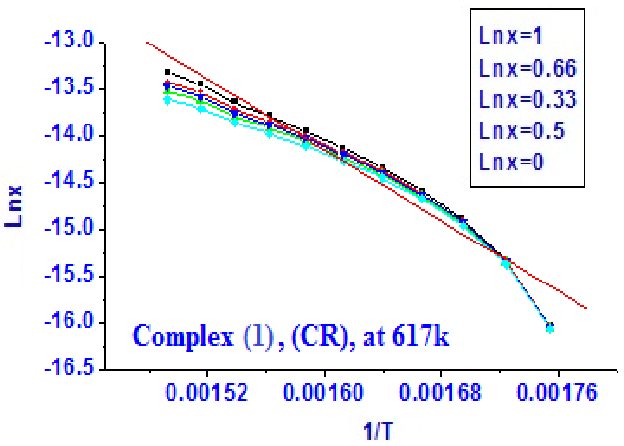
Lnx=1
Lnx=0.66
Lnx=0.33
Lnx=0.5
Lnx=0
Lnx
1/T
Complex (1), (CR), at 617k

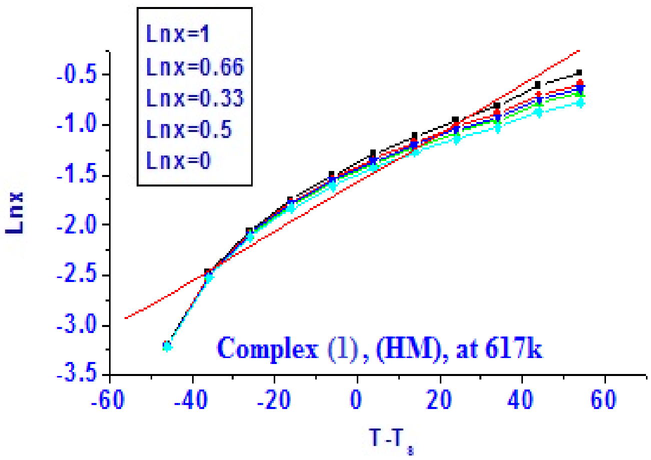
Lnx=1
Lnx=0.66
Lnx=0.33
Lnx=0.5
Lnx=0
Lnx
T-Tg
Complex (1), (HM), at 617k

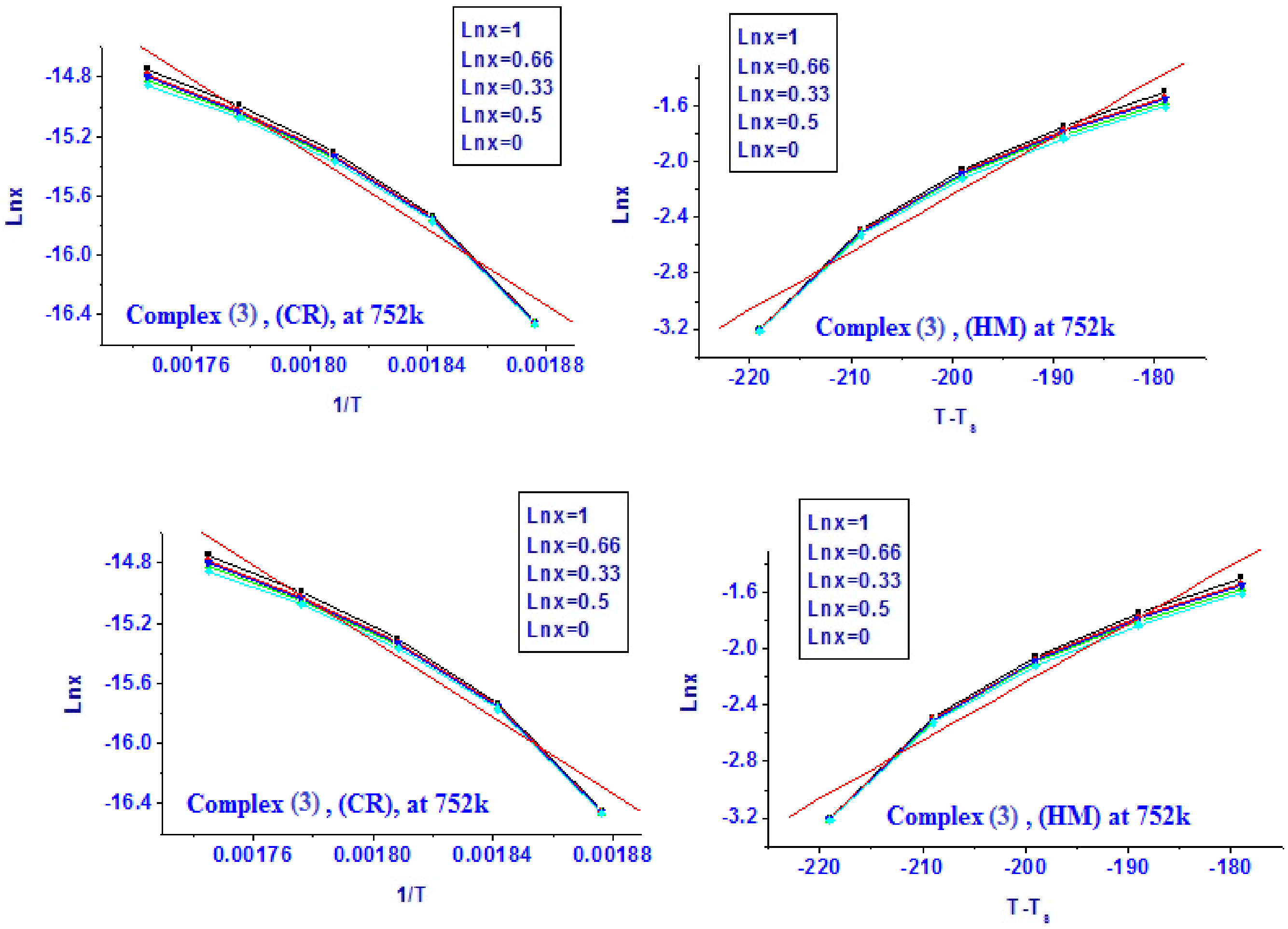

Figure S6: The diagrams of kinetic parameters for CETZ.2HCl, Phen.H_2O and their metal complexes.

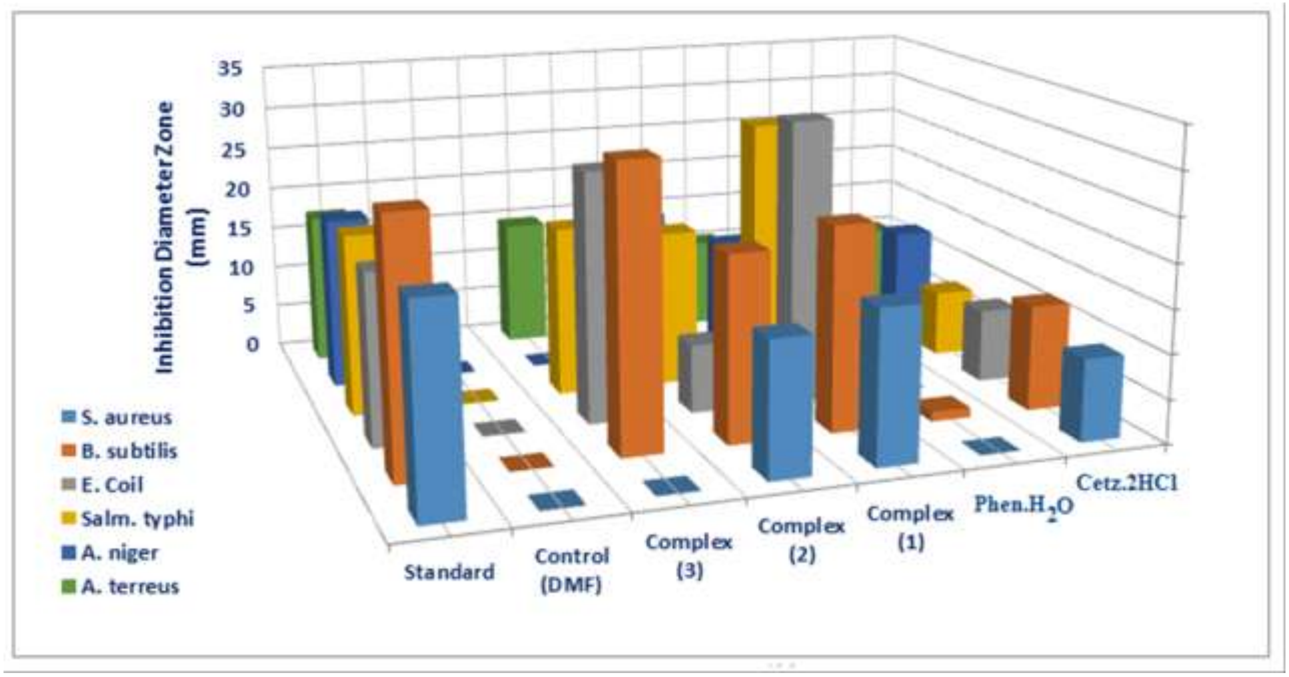

Figure S7: Statistical representation for biological activity for CETZ.2HCl, Phen.H_2O and their chelates.

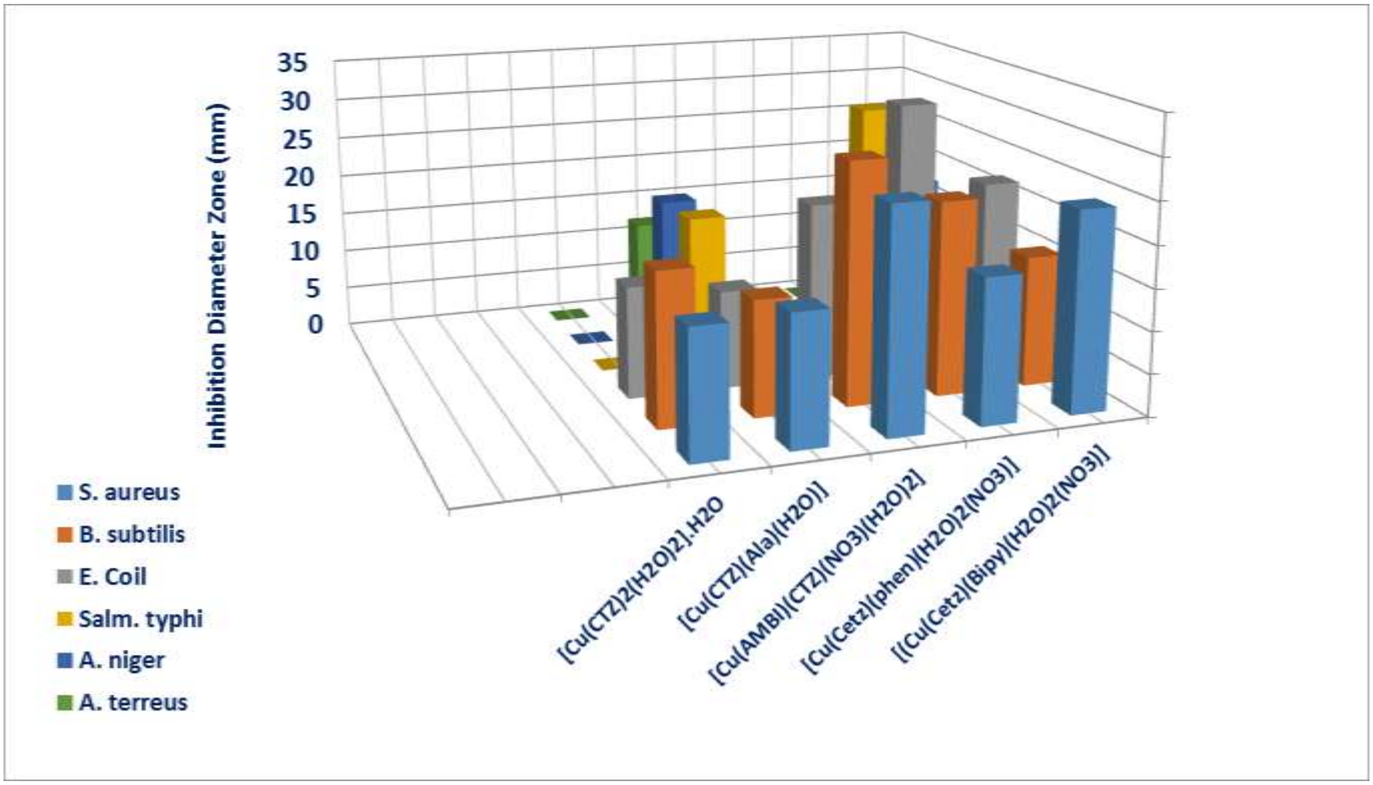

Figure S8: Statistical representation of biological comparison between Cu(II) complex in our complexes and some previous works.

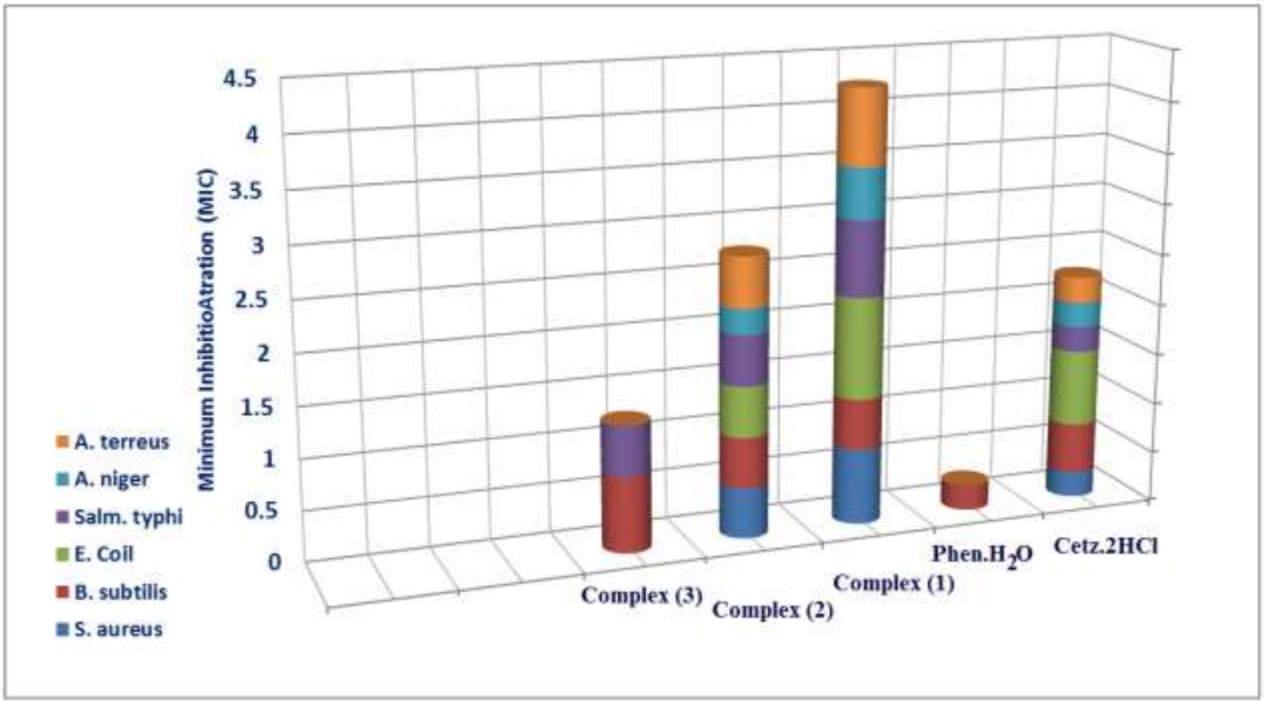

Figure S9: MIC for the sensitive bacteria and fungi for CETZ.2HCl, Phen.H_2O and their metal complexes.

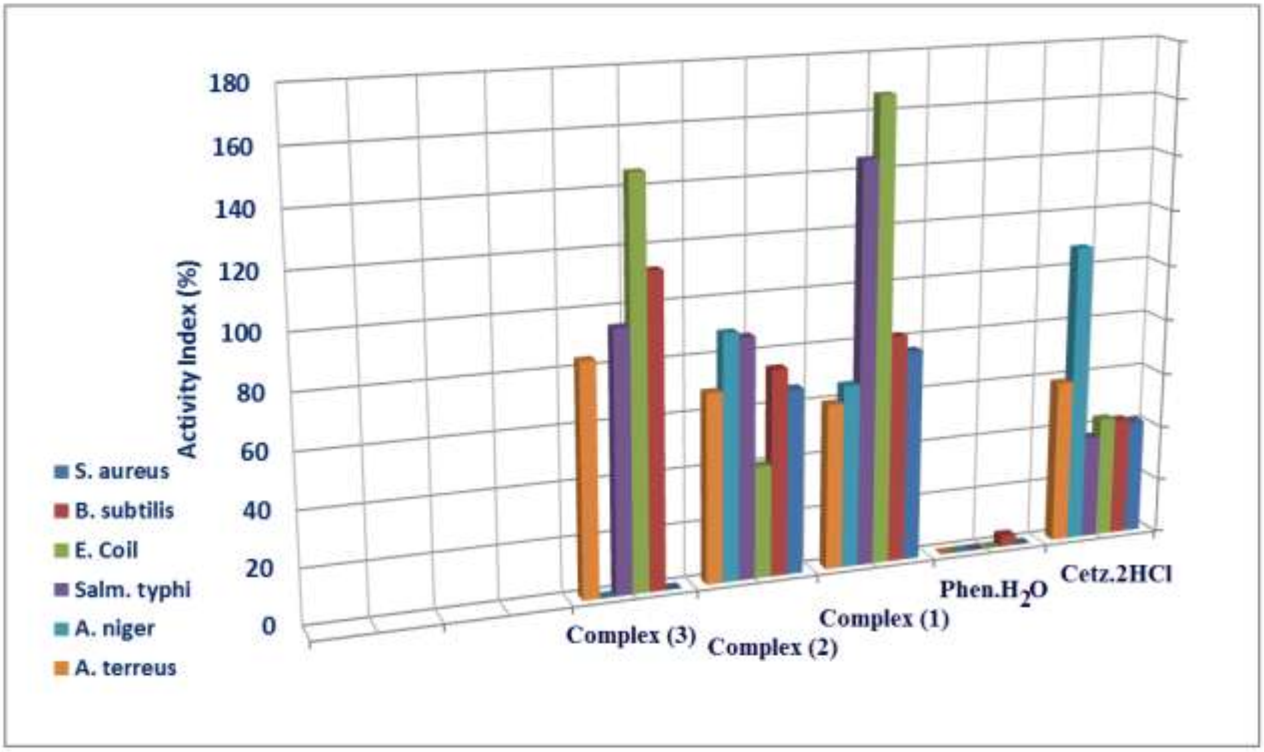

Figure S10: Activity index % for CETZ.2HCl, Phen.H_2O and their metal complexes.

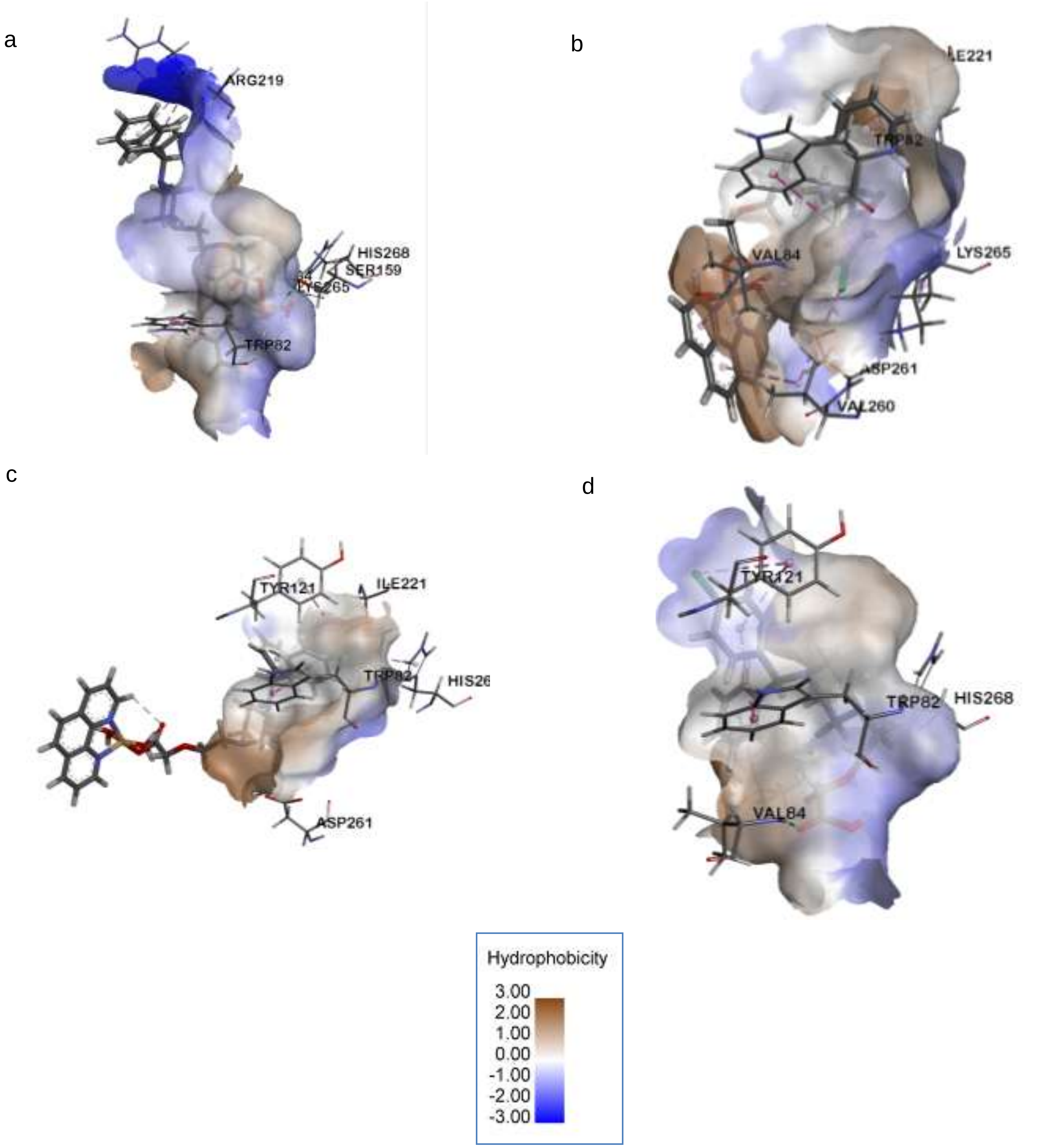

Figure S11: 3D binding mode and residues involved in the recognition of (a) Cu(II) complex, (b) Zn(II) complex, (c) Cd(II) complex, and (d) CETZ docked and minimized in the SOD binding pocket.

Table S1. Comparing mice administered CETZ.2HCl antihistamine and its metal-derived complexes with those not receiving the drug in terms of serum lipase enzyme and lipid profile levels.

Animal groups	Enzyme	Lipid profile (mean ± SD)			
	**L. lipase (U/L)**	**Total Chol. (mg/dl)**	**Trig (mg/dl)**	**HDL (mg/dl)**	**LDL (mg/dl)**
untreated (controls) [**ref.**]	66.88 ± 5.3	220.38± 30.25	140.38 ±14.47	90.88 ± 7.24	81.25 ± 9.3
Treated with **CETZ.2HCl**	104.88± 26.5**	230.38± 25.56NS	80.5±10.18**	85.25±6.23NS	93.25±8.15*
Treated with **Cu-CETZ Complex**	127.88±11.72**	138.88±10.92**	77.25±9.3**	80.75±8.26 *	60.5±5.78**
Treated with **Zn-CETZ Complex**	108.88±20.0**	155.5 ±16.01**	70.63±6.05**	115.75±13.32**	71.63±6.5*
Treated with **Cd-CETZ Complex**	52.38±9.6**	283.75 ± 43.16**	188.75 ±18.09**	44.63±12.92**	89.25 ±9.45*

Qualitative data was analysed using the Chi-square test or Fisher's Exact test (median (N-value)± IQR), while quantitative (normally distributed) data was expressed in (mean (N-value) ± SD) and was compared using t-student test. As compared to controls, the P <0.05 considered statistically significant, P >0.05 considered non significant, P <0.01 P highly significant, IQR: interquartile range , SD:standard deviation

Student-*t*-test analyses were performed between drug-treated groups versus controls, considering significant differences as ** highly significant (*P <0.01*), * significant (*P < 0.05*) and non-significant (*NS, P > 0.05*).

Table S2 The mean Lipase activity (U/L), docking scores[a] and type of binding interactions of (1), (2), (3) complexes and the reference compound (Cetirizine).

Complexes	Mean L.Lipase activity (U/L)	Binding energy (Kcal/mol) (docking score)	Type of binding interactions
**(1)**	127.88	-12.4	• H-bonds with Ser159 and His268<br>• Hydrophilic interactions with Trp82<br>• π-πT-shapedinteractions with Trp82<br>• π-alkyl interactions with Arg219, Val264 and Lys265
**(2)**	108.88	-10.8	• H-bonds with Asp261<br>• π-π T-shaped interactions with Trp82<br>• π-alkyl interactions with Val84, Ile221, Val260 and Lys265
**(3)**	52.38	-5.5	• Hydrophilic interactions with Asp261<br>• π-π stacking interactions with Trp82<br>• π-sigmainteractions withIle221<br>• π-alkyl interactions with Tyr121and His268
**CETZ.2HCl**	104.88	-9.6	• H-bonds with Val84<br>• Hydrophilic interactions with Trp82 and His268<br>• π-π stacking interactions with Trp82 and Tyr121<br>• π-alkyl interactions with Val264

- Docking was carried out against the lipase enzyme pocket (PDB code ID: 6E7K)
- [a]More negative score refers to better cabability of a molecule to dock with the target and make more desirable interactions.

TABLE S3 Equilibrium geometric parameters, bond lengths (Å), bond angles (˚), dihedral angles (˚), total energy (kcal/mol) and dipole moment of CETZ.2HCl by using DFT calculations.

Bond length (Å)							
C1-C2	1.342	C5-C8	1.523	C10-C15	1.346	N9-C20	1.457
C2-C3	1.343	C8-C10	1.522	C8-N9	1.461	N18-C21	1.451
C3-C4	1.342	C10-C11	1.346	C16-N9	1.453	C21-C22	1.533
C4-C5	1.346	C11-C12	1.342	C16-C17	1.535	C22-O23	1.413
C5-C6	1.345	C12-C13	1.341	C17-N18	1.448	C24-O23	1.411
C1-C6	1.343	C13-C14	1.344	C19-N18	1.449	C24-C25	1.514
C2-Cl7	1.726	C14-C15	1.343	C19-C20	1.538	C25-O26	1.206
						C25-O27	1.332
**Bond angle (˚)**							
C8C5C4	120.72	C8N9C16	115.51	C22O23C24	113.45		
C8C5C6	121.28	C8N9C20	113.64	O23C24C25	109.38		
C5C8C10	106.46	C21N18C17	68.31	C24C25O26	126.56		
C10C8N9	112.43	C21N18C19	117.35	C24C25O27	110.85		
C5C8N9	113.42	N18C21C22	117.92	C8C10C15	121.83		
C8C10C11	120.19	C21C22O23	107.24				
**Dihedral angles (˚)**							
C10C8C5C6	68.52	C21N18C19C20	94.99				
C1510C8C5	-93.23	O23C22C21N18	-179.74				
N9C8C10C11	-150.68	O26C25C24O23	127.01				
C16N9C8C5	-45.41	C11C10C8C5	84.84				
C21N18C17C16	-93.16	N9C8C5C6	-55.35				
C22C21N18C17	66.39	C16N9C8C10	-165.72				
C25C24O23C22	-178.44	C8N9C16O17	-71.48				
C10C8C5C4	-110.36	C22C21N18C19	-71.98				
N9C8C5C4	125.77	C24O23C22C21	173.87				
N9C8C10C15	31.25	O27C25C24O23	-57.02				
C8N9C20C19	74.51						
Total energy, kcal/mol		-15813.849					
Dipole moment, D		4.26					

Table S4: Equilibrium geometric parameters bond lengths (Å), bond angles (˚), dihedral angles (˚), Total energy (k cal/mol) and Dipole moment of the studied complexes by using DFT calculations.

Bond lengths/ Å	Cu(II)	Zn(II)	Cd(II)
M-N1	2.291	2.019	2.461
M-N4	2.292	2.016	2.465
M-O5	1.901	2.069	2.327
M-O6	2.238	2.157	2.241
M-O7	2.244	2.149	2.237
M-X8	2.437	2.260	2.337
N1-C2	1.264	1.265	1.267
C3-N4	1.263	1.261	1.265
C2-C3	1.338	1.335	1.344
N1-M-N4	78.93	77.02	70.65
N1-M-O5	98.54	164.66	158.75
N1-M-O6	84.74	88.96	79.56
N1-M-O7	167.45	95.74	105.78
N1-M-X8	95.35	94.56	81.11
N4-M-O5	82.43	92.76	96.07
N4-M-O6	96.61	90.54	94.51
N4-M-O7	96.47	170.82	164.07
N4-M-X8	173.62	92.87	84.79
O5-M-O6	176.31	81.36	85.25
O5-M-O7	92.36	93.14	91.39
O5-M-X8	95.76	97.39	115.01
O6-M-O7	84.20	83.41	100.13
O6-M-X8	85.59	176.43	159.72
O7-M-X8	89.70	93.34	72.29
Total energy, k cal/mol	-310833.820	-249442.317	-270148.039
Dipole moment, D	24.691	14.19	17.381

(X= O8 in all complexes except in case of Zn complex X= Cl8)

Table S5: Calculated charges on donating sites and energy values (HOMO, LUMO, Energy gap ΔE/eV, hardness (η), global softness (S), electro negativity (χ), absolute softness (σ), chemical potential (Pi), global electrophilicity (ω) and additional electronic charge (ΔN_{max}) of CETZ.2HCl, Phen.H_2O and thier complexes by using DFT calculations.

Parameters	**CETZ.2HCl**	**Phen.H_2O**	Cu(II)	Zn(II)	Cd(II)
M	-	-	0.008	0.120	0.326
N1	-	-0.209	-0.043	-0.077	-0.110
N4	-	-0.213	0.126	-0.062	-0.111
O5	-0.379	-	-0.099	-0.474	-0.488
O6	-	-	-0.340	-0.344	-0.364
O7	-	-	-0.290	-0.298	-0.330
X8	-	-	-0.414	-0.577	-0.514
HOMO, H	-0.356	-0.396	-0.329	-0.337	-0.337
LUMO, L	-0.198	-0.153	-0.308	-0.239	-0.237
I = -H	0.356	0.396	0.329	0.337	0.337
A = -L	0.198	0.153	0.308	0.239	0.237
ΔE = L-H	0.158	0.243	0.021	0.098	0.100
η = (I-A)/2	0.079	0.122	0.0105	0.049	0.050
χ = -(H-L/2)	0.277	0.275	0.3185	0.288	0.287
σ = 1/ η	12.658	8.197	95.238	20.408	20.000
S = 1/2 η	6.329	4.098	47.619	10.204	10.000
Pi = - χ	-0.277	-0.275	-0.3185	-0.288	-0.287
$\omega = (Pi)^2/2\eta$	0.486	0.309	0.910	0.846	0.824
$\Delta N_{max} = \chi/\eta$	3.506	2.254	30.333	5.878	5.740

(I) is ionization energy
(A) is an electron affinity
(X8 = O in all complexes except in Zn complex X8= Cl)

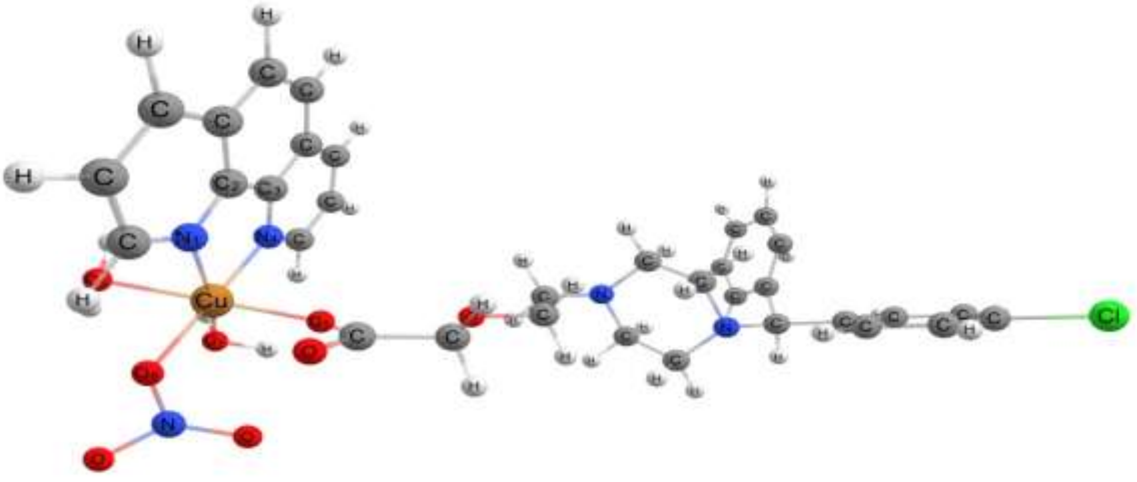

Scheme S1: DFT-Optimized geometrical structure of Cu(II) complex.

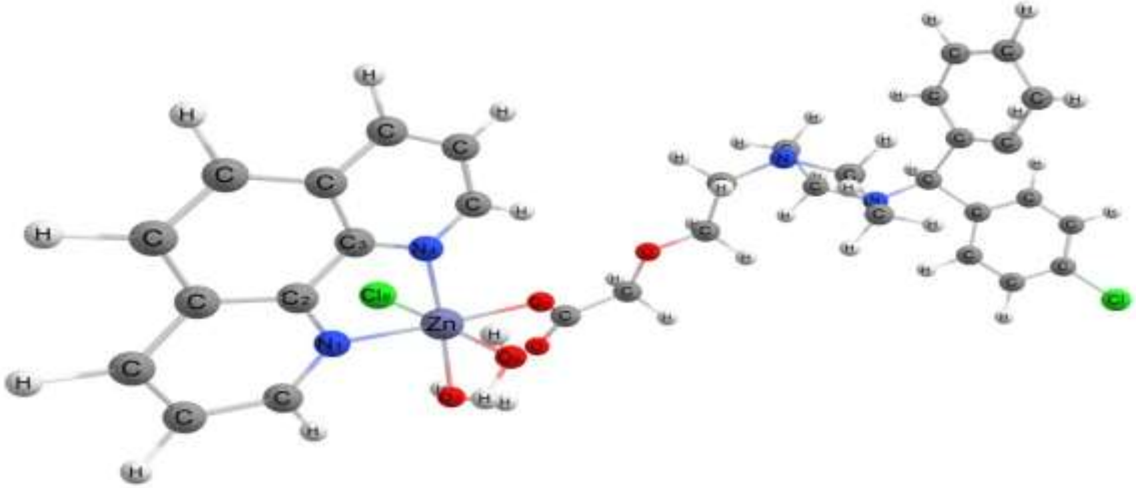

Scheme S2: DFT-Optimized geometrical structure of Zn(II) complex.

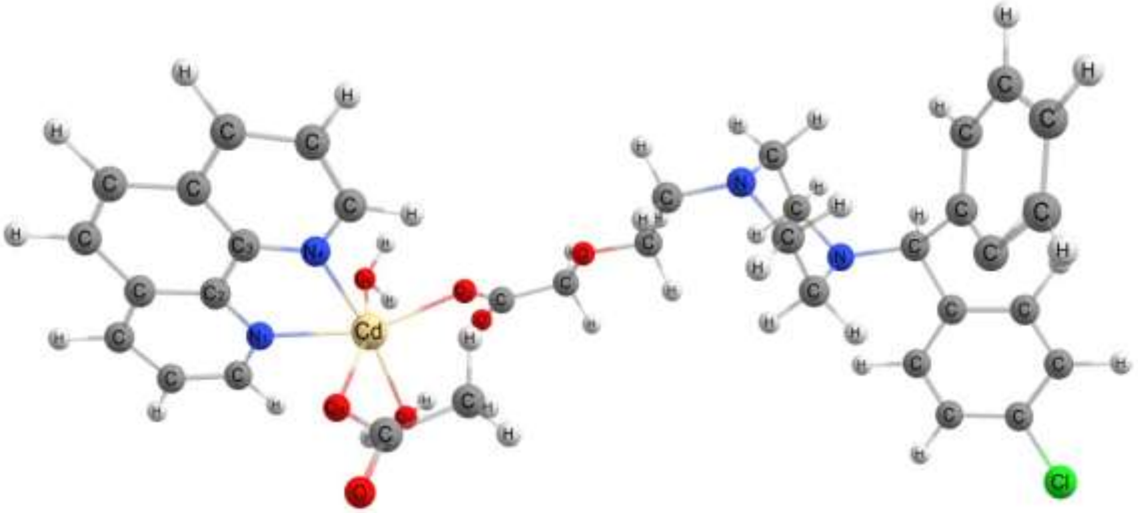

Scheme S3: DFT-Optimized geometrical structure of Cd(II) complex.
